# Data-driven analysis to identify prognostic immune-related biomarkers in BRAF mutated cutaneous melanoma microenvironment

**DOI:** 10.3389/fgene.2022.1081418

**Published:** 2022-11-30

**Authors:** Biao Huang, Wenxing Su, Daojiang Yu

**Affiliations:** ^1^ Department of Plastic and Burn Surgery, The Second Affiliated Hospital of Chengdu Medical College, China National Nuclear Corporation 416 Hospital, Chengdu, China; ^2^ Department of Clinical Medicine, Chengdu Medical College, Chengdu, China; ^3^ West China School of Basic Medical Sciences and Forensic Medicine, Sichuan University, Chengdu, China

**Keywords:** BRAF mutated melanoma, TCGA, immune cells, prognosis, microenvironment

## Abstract

Skin cutaneous melanoma is one of the deadly diseases, and more than 50% of the patients have BRAF gene mutations. Evidence suggests that oncogenic BRAF modulates the immune system’s ability to recognize SKCM cells. Due to the complexity of the tumor microenvironment (TME) and a lack of a rational mechanistic basis, it is urgent to investigate the immune infiltration and identify prognostic biomarkers in BRAF mutated SKCM patients. Multiple methods including ESTIMATE algorithm, differential gene analysis, prognostic analysis and immune infiltration analysis were performed to investigate the tumor microenvironment. Based on the patient’s immune score and stromal score, immune-related genes DEGs were identified. Functional analysis revealed that these genes were mainly enriched in biological processes such as immune response, defense response and positive regulation of immune system. Furthermore, we analyzed the immune infiltrating cell components of BRAF mutated patients and revealed 4 hub genes associated with overall survival time. Several cells (Monocyte, Macrophage and Gamma delta cells) have been found to be significantly decreased in immune-high BRAF mutated SKCM group. While CD4^+^T, CD8^+^T, CD4 naïve, Tr1, Th2 and many T cell subsets were significantly increased in immune-high group. These immune cells and genes were closely related to each other. This study revealed that the dysregulation of immune function and immune cells may contribute to the poor outcomes of BRAF mutated patients. It is of great significance to our further understanding of the TME and immune dysfunction in BRAF mutated SKCM.

## Introduction

Skin cutaneous melanoma (SKCM) is one of the most aggressive malignancies, causing about 80% of deaths in skin cancer (Schadendorf et al., 2018). Nearly 50% of cutaneous melanoma harbor activating V600E mutations in BRAF, which is considered a prognostic indicator of tumor proliferation, metastasis, recurrence as well as an effective target for SKCM treatment. The major factor limiting the clinical benefit of BRAF inhibitor are short response duration, off-target effect and drug resistance (Ribas et al., 2019a). There is also evidence that oncogenic BRAF can modulate the ability of the immune system to recognize SKCM cells. Activating mutations in the BRAF gene activate the mitogen-activated protein kinase (MAPK) pathway, which contributes to immune escape by recruiting regulatory T cells, reducing antigen presentation, and inhibiting the release of IFN-γ and TNF-α (Ascierto and Dummer, 2018). A series of immunotherapy strategies such as anti-PD-1, anti-CTLA4 and MAGE-A3 have been applied in SKCM and result in improvement in patient survival (Bajor et al., 2018). In addition, the combination of BRAF inhibitors and anti-PD-1 has shown significant improvement in SKCM treatment response (Ascierto et al., 2019a). These results suggest that we may be able to improve the survival outcome of BRAF mutated patients by regulating their immune response and tumor microenvironment. The tumor microenvironment (TME) consists of a variety of immune cells and stromal cells, including fibroblasts, endothelial cells, extracellular matrix, cytokines, chemokines and receptors (Vigneron, 2015). Due to the complexity of TME and a lack of a rational mechanistic basis, it is urgent to investigate the tumor microenvironment and identify prognostic biomarkers in BRAF mutated SKCM patients (Gnanendran et al., 2020).

ESTIMATE algorithms have been developed to calculate tumor purity in various cancers based on the specific gene expression signature of immune and stromal cells (Yoshihara et al., 2013). In this current work, we applied the expression data of BRAF mutated SKCM cohorts and ESTIMATE algorithm to extract a list of tumor microenvironment associated genes. Most of the genes were found to be related to better survival outcomes in BRAF mutated SKCM patients. Importantly, we estimated the proportion of immune cells based on gene expression profiling in BRAF mutated samples. Finally, we identified 4 hub genes associated with prognosis and immune cell infiltration in BRAF patients.

## Materials and methods

### Database of BRAF mutated SKCM patients

Transcriptional data of BRAF mutated SKCM patients (n = 240)was downloaded from the TCGA (https://tcga-data.nci.nih.gov/tcga/). In addition, their age, sex, tumor stage and survival information were obtained from the clinical documents in TCGA database (Tomczak et al., 2015). Statistical information of BRAF mutated SKCM patients was downloaded from Tumor Immune Estimation Resource dataset (Li et al., 2017). As a validation dataset, transcriptional data of SKCM patients (n = 131) was download from Gene Expression Omnibus (GEO) (Barrett et al., 2013). Screening criteria include: 1) the clinical diagnosis was skin cutaneous melanoma and 2) detection of BRAF mutation character. The discharge criteria include: 1) clinical data without survival time and outcome, and 2) datasets with small sample sizes (n < 50). Finally, the datasets were eligible: accession number GSE22153 (n = 131).

### Calculation of immune and stromal scores

We used the ESTIMATE method to calculate the immune score and stromal score for each patient (Yoshihara et al., 2013). It is widely used to characterize the composition of infiltrating stromal cells and immune cells in tumor tissues.

### Analysis of DEGs

BRAF mutated SKCM patients were ranked and divided into top and bottom halves (high vs. low score groups) based on their immune scores. Similarly, based on the stromal scores, the SKCM samples were grouped into high-stromal group and low-stromal group. Differentially expressed genes (DEGs) between the high-immunity/high-stromal group and low-immunity/low-stromal group were identified using the “limma” package in R software. |log(Fold change)| > 2, *p* < 0.05 and FDR<0.05 were set as the cutoffs.

### Survival analysis

Overall survival data collected from each BRAF mutated SKCM patient were used to perform Kaplan-Meier analysis to explore the prognostic genes among the above DEGs. Patients with a given gene expression above 50% were designated as the high-expression group, while those with gene expression below 50% were designated as the low-expression group. Using log-rank method to test significance. The *p* value <0.01 was set as the cut-off value. Then, based on the survival data from GSE22153, we verified the prognostic value of prognostic genes in TCGA. The validated prognostic genes were used for subsequent protein-protein interaction analysis.

### Function annotation

In order to reveal the function of DEGs and module genes, function annotation and Genome (KEGG) pathway enrichment analysis were performed using DAVID (Huang et al., 2007). FDR< 0.05 and *p* < 0.01 were set as the cut-off.

### Protein-protein interaction network and model analysis

Evaluation of the protein-protein interaction network coded by validated prognostic genes was constructed by STRING (Szklarczyk et al., 2015), and their co-expression network was displayed by Cytoscape (Shannon et al., 2003). Then, the plugin Molecular Complex Detection (MCODE) was applied to identify the module genes that interact most closely.

### Hub genes selection, validation and their co-expression network

Hub genes were obtained in this study by using Cytohubba plugin. The top ten genes in our PPI network were calculated based on six algorithms (MCC, MNC, EPC, Closeness, Radiality, Degree) at the same time. In addition, the intersection genes contained in the results of the six algorithms are screened out by upset calculation. Then, we used these genes as hub genes for further analysis. By using the Genemania database, we constructed the co-expression network of these hub genes and investigated their function (Warde-Farley et al., 2010). We used GSE22153 data to verify the mRNA expression of hub genes. To further validate our findings, we searched the Human Protein Atlas (https://www.proteinatlas.org/) website for the immunohistochemical (IHC) staining results of nine hub genes in normal skin and tumor tissue.

### Immune cell components of BRAF mutated SKCM patients

To quantify the immune cell components of BRAF mutated SKCM patients, the expression data of patients were applied to calculate the composition of infiltrating immune cells by using ImmuCellAI algorithm (Racle et al., 2017). Based on the transcribed data of tumor tissue, the deconvolution algorithm can well reflect the infiltration and composition of immune cells. In this article, 24 kinds of immune cells such as neutrophils and NKT were calculated using ImmuCellAI algorithm. In addition, we compared the difference of immune cells between immune-high group and immune-low group using *t*-test. Moreover, the spearman correlation coefficient was calculated between immune cells and hub genes.

### Statistical analysis

Analysis of DEGs, function annotation, survival analysis, ROC curves were all performed and visualized in R software. *t*-test was used to calculate the significant difference of immune and stromal scores among different AJCC stages and Breslow depth. *p*-values<0.05 were considered a statistically significant cut-off in all tests.

## Results

### Clinical information of BRAF mutated cutaneous melanoma patients

According to the inclusion criteria, 240 BRAF mutated SKCM patients from TCGA and 131 SKCM patients from GSE22153 (n = 131) were collected finally. In our study, the clinicopathological characteristics of BRAF mutated SKCM patients were shown in Table 1.

**TABLE 1:** Clinicopathological characteristics of SKCM patients.

Characteristics	GEO cohort (N = 131)	TCGA cohort (N = 240)	*p* Value (high-immune vs. low-immune)	*p* Value (high-stromal vs. low-stromal)
N (%)				
Age				
≤60 years	85 (65.9)	149 (62.1)	ns	ns
>60 years	46 (34.1)	91 (37.9)	ns	ns
Gender				
Male	90 (68.7)	148 (61.7)	ns	ns
Female	41 (31.3)	92 (38.3)	ns	ns
Clark level				
I	18 (13.9)	82 (34.1)	<0.05	ns
II	36 (26.8)	9 (3.7)	ns	ns
III–IV	66 (50.7)	137 (57.1)	<0.01	<0.05
V	11 (8.6)	12 (5.1)	ns	ns
Breslow depth(mm)				
≤0.75	21 (16.1)	85 (35.4)	<0.01	<0.05
0.76–1.50	42 (32.2)	38 (15.8)	ns	ns
1.51–4.00	55 (41.9)	62 (25.8)	<0.05	ns
>4.00	13 (9.8)	55 (23.0)	ns	<0.05
pT stage				
T1-T2	NA	124 (51.7)	<0.01	<0.05
T3-T4	NA	116 (48.3)	<0.05	<0.05
pN stage				
N0	NA	150 (62.5)	<0.05	ns
N1	NA	43 (17.9)	ns	ns
N2	NA	47 (19.6)	ns	ns
pM stage				
M0	NA	226 (94.2)	ns	ns
M1	NA	14 (5.8)	ns	ns
Pathologic stage				
I- II	38 (28.9)	144 (60.0)	<0.05	<0.05
III-IV	93 (71.1)	96 (40.0)	<0.05	<0.05
Persistent distant metastasis				
No	46 (35.0)	54 (22.5)	ns	ns
Yes	85 (65.0)	186 (77.5)	ns	ns

SKCM, skin cutaneous melanoma; TCGA, the cancer genome atlas; GEO, the gene expression omnibus; NA, not available; ns, not significant.

### Immune and stromal score are closely related to the prognosis of SKCM

**FIGURE 1:**
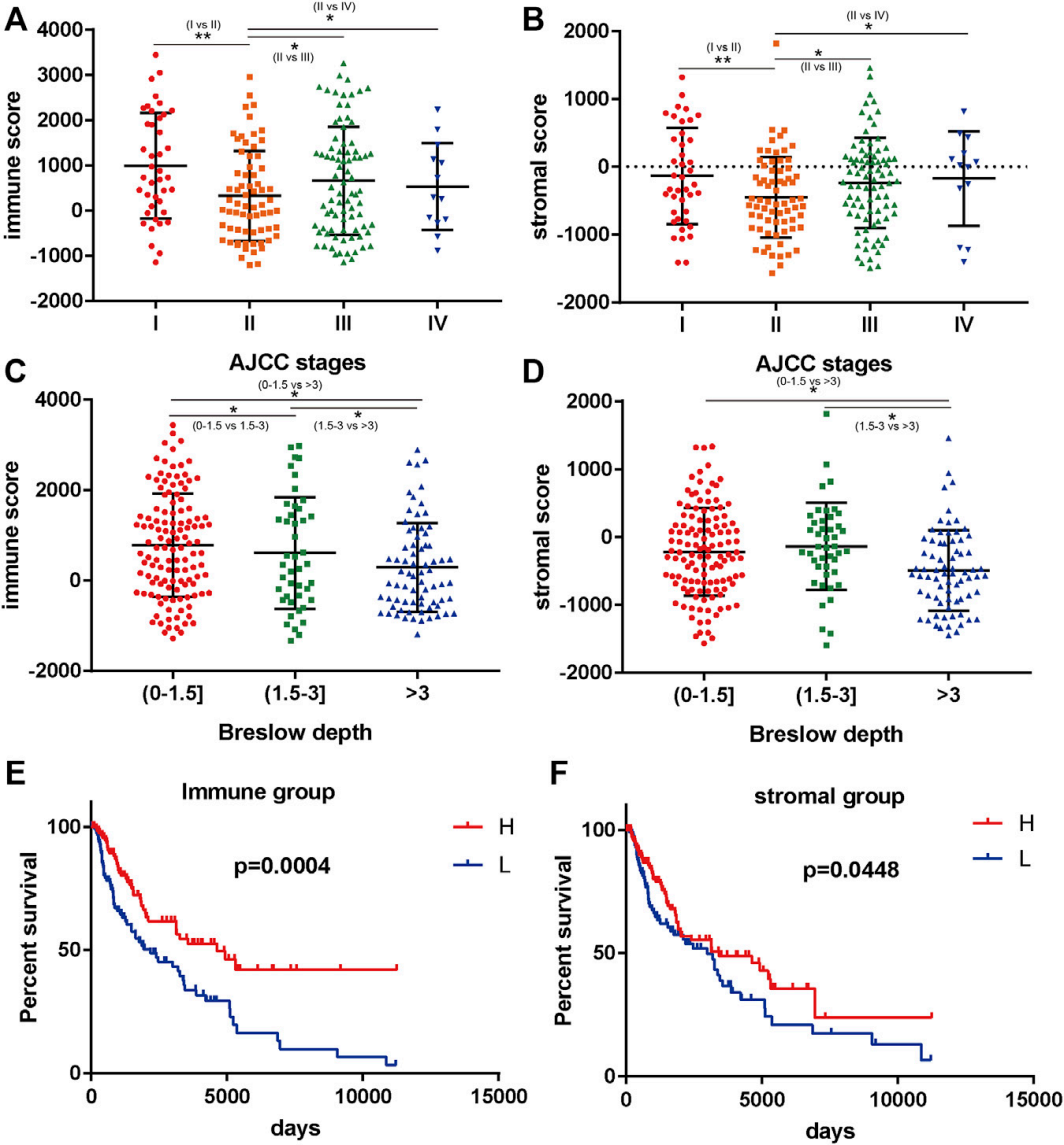
Immune scores and stromal scores are closely associated with BRAF mutated melanoma prognosis. **(A**,**B)** the correlation between immune/stromal score and AJCC stage. **(C**,**D)** the correlation between immune/stromal score and Breslow depth. **(E**,**F)** high immune scores and stromal scores were associated with longer survival (*p* < 0.05). *p < 0.05; **p < 0.01; ***p < 0.001.

According to the ESTIMATE results, the immune score of 240 BRAF mutated SKCM patients (TCGA) ranged from -1133.65 to 3441.88. In addition, stromal score of BRAF mutated SKCM patients ranged from -1597.24 to 1817.91. We evaluated the correlation between immune, stromal score and clinicopathological characteristics of SKCM patients. In Figures 1C,D, we found that when the Breslow depth >3 mm, the immune score was significantly lower than that in 0–1.5 mm group and 1.5–3 mm group (*p* < 0.05). Similar results could be found in the stromal score. We also found that there was a significant correlation between immune score, stromal score and AJCC stage (American Joint Committee on Cancer) of SKCM (*p* < 0.05, Figures 1A,B). When comparing the immune and stromal scores of different AJCC stages, significant differences could be observed between several groups (I vs. II, II vs. III and II vs. IV).

We further analyzed the relationship between immune and stromal scores and the prognosis of SKCM. A total of 240 BRAF mutated SKCM patients were ranked according to their immune scores and stromal scores. Then, we divided the 240 SKCM cases into top (n = 120) and bottom halves (n = 120) based on their scores. Among them, high level of immune score and stromal score were found significantly associated with longer overall survival (Figures 1E,F, *p* < 0.05).

### Differentially expressed genes between high vs. low group and their function annotation

**FIGURE 2:**
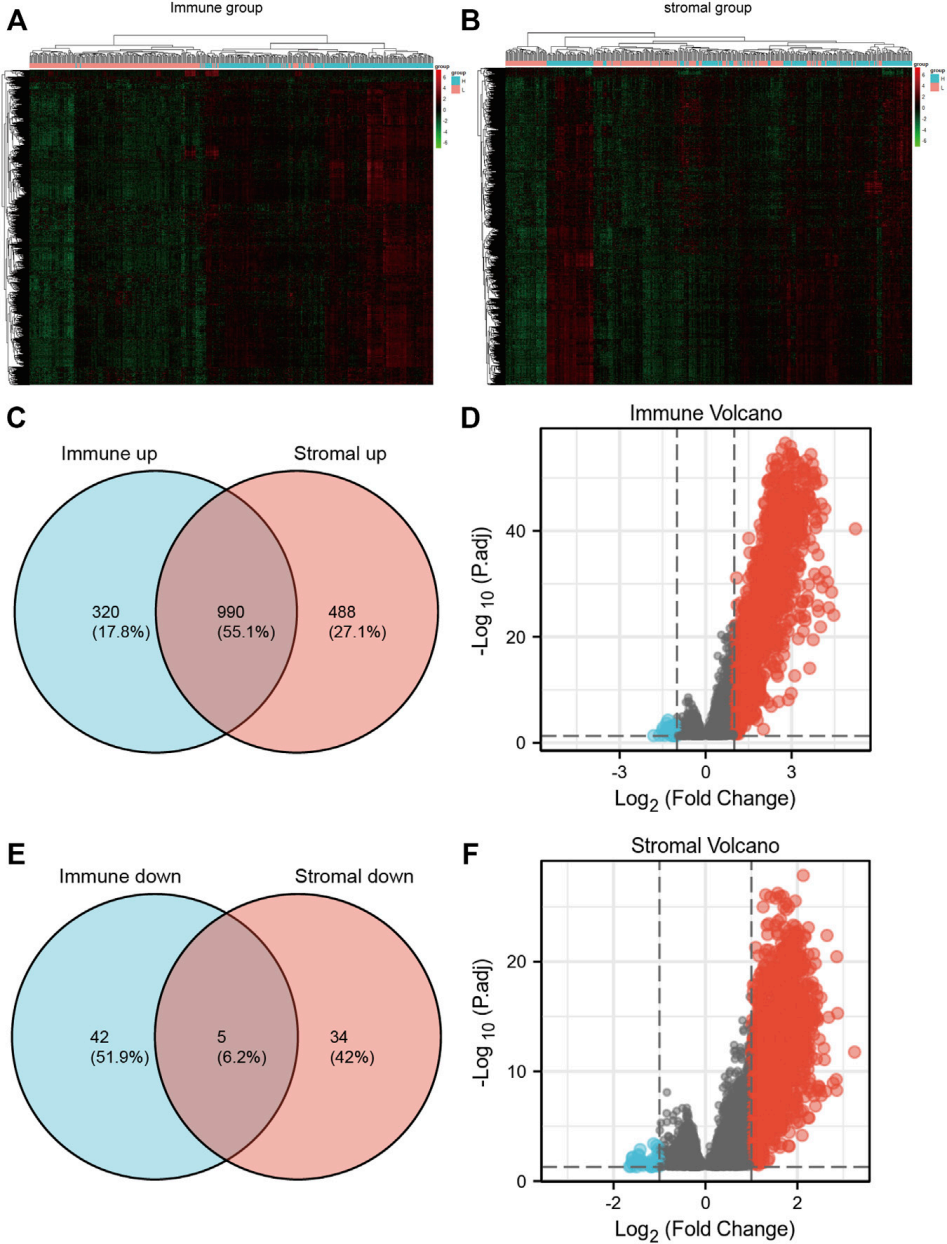
**(A**,**B)** heatmaps of gene expression profiles of samples between high immune/stromal and low immune/stromal groups. **(C**,**D)** the up-regulated and down-regulated overlapped DEGs. **(E**,**F)** volcano plot of immune and stromal DEGs.

In view of the fact that immune and stromal scores were closely related to SKCM prognosis. Differentially expressed genes between high vs. low group were identified. The heatmap of gene expression showed a significant difference between immune high and immune low group. Similar results could be found between stromal high and stromal low group (Figures 2A,B). As a result, there were 1310 genes upregulated and 47 genes downregulated between high immune group and the low group (|logFoldChange| >2; *p* < 0.05). Additionally, there were 1478 genes upregulated and 39 genes downregulated between high stromal group and low group (|logFoldChange| >2; *p* < 0.05) (Figures 2E,F).

**FIGURE 3:**
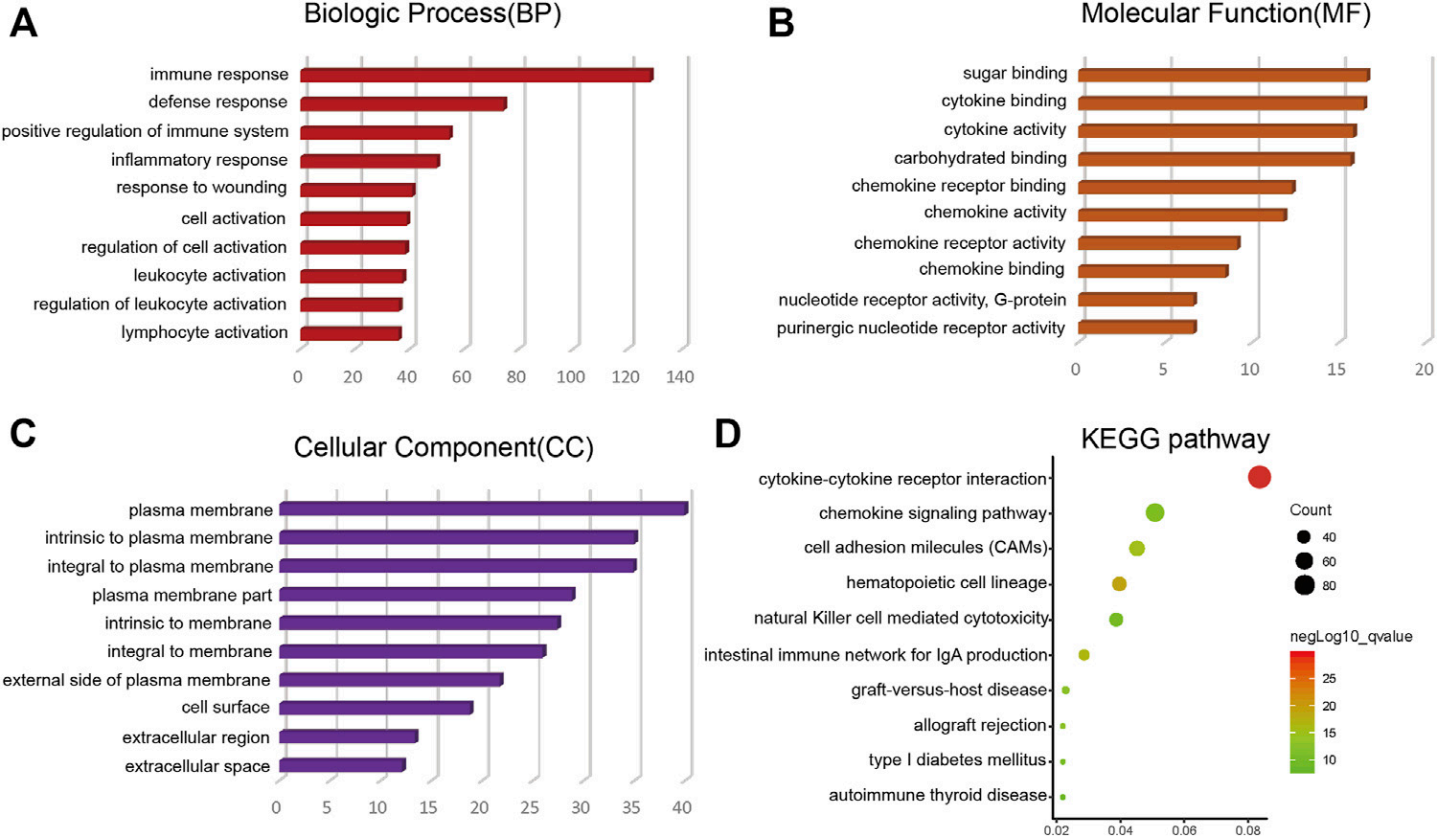
Top 10 GO terms (BP, MF, CC) and KEGG analysis of overlap DEGs (*p* < 0.01). **(A)** BP results. **(B)** MF results. **(C)** CC results. **(D)** KEGG pathways. GO, Gene Ontology; BP, biological process; MF, molecular function; CC, cellular component; KEGG, Kyoto Encyclopedia of Genes and Genomes.

Through the intersection of the Venn diagram, there were 990 overlap genes which both upregulated in the immune and stromal groups (Figure 2C). There were only 5 genes which both downregulated in the immune and stromal groups (Figure 2D). Therefore, the overlapped 990 genes were selected for further analysis. Function annotation has been carried out among the 990 overlap genes (*p* < 0.01; FDR<0.01). BP category suggested that immune response, defense response, inflammatory response, positive regulation of immune system and leukocyte activation were important process of the overlap genes (Figure 3A). As expect, MF results indicated that these upregulated overlap genes were mostly involved in sugar binding, cytokine activity, chemokine receptor binding and chemokine activity (Figure 3B). The plasma membrane, intrinsic to the plasma membrane, plasma membrane part items in CC category, indicating 990 overlapped genes play their roles in the plasma membrane (Figure 3C). In addition, chemokine signaling pathway, cytokine-cytokine receptor interaction, and cell adhesion molecules were important pathways of the overlapped gene network (Figure 3D).

### Correlation of expression of individual DEGs in overall survival

**FIGURE 4:**
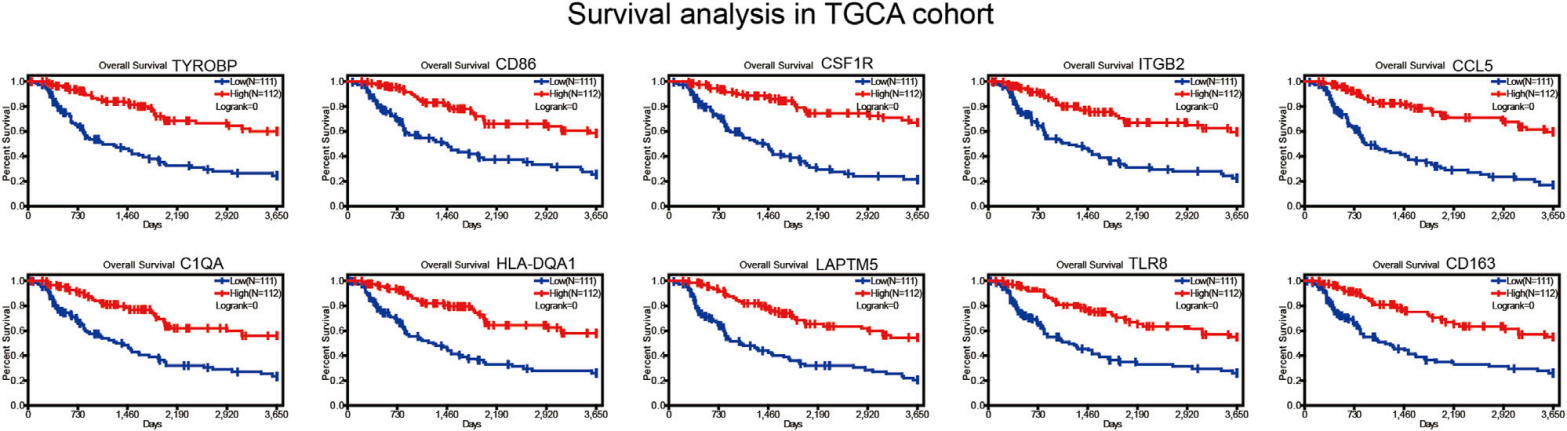
Survival curves for immune‐related genes in TCGA cohort.

The overlap 990 upregulated genes were used to identify prognostic genes through survival analysis. Subsequently, 755 genes (76%) were found correlated with longer overall survival time (*p* < 0.01, Figure 4, Supplementary Table S1). These genes were considered immune-related prognostic genes for further study.

### Survival verification in GEO cohort

**FIGURE 5:**
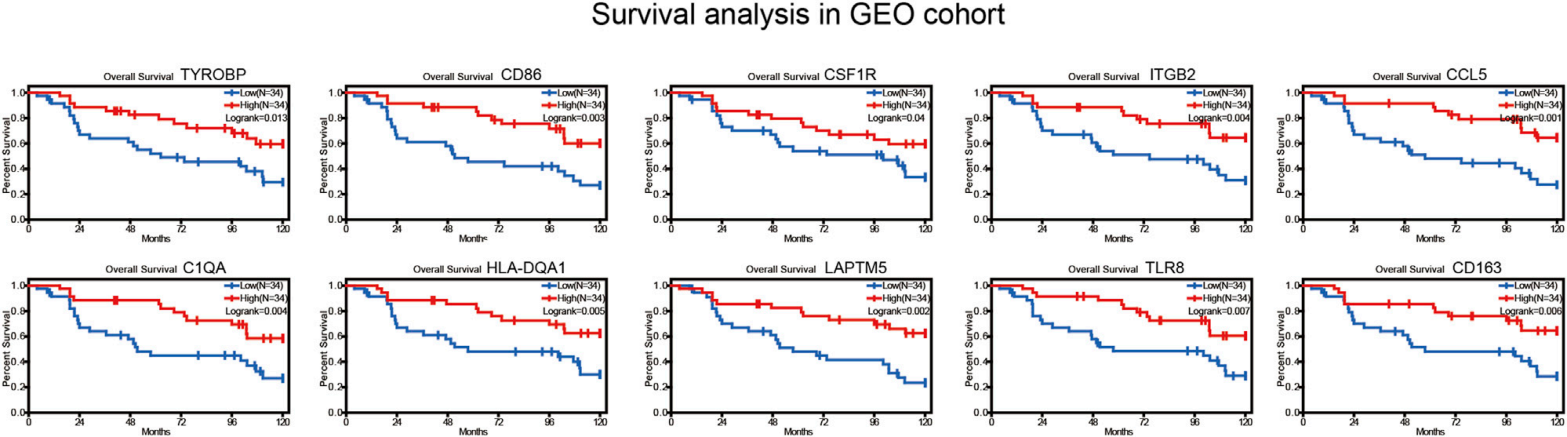
Survival curves for immune‐related genes in GEO cohort.

We collected BRAF mutated SKCM patients from GSE22153 from GEO database. Based on their overall survival data, 755 prognostic genes were selected to further verify their survival value. As a result, a total of 107 genes out of 755 identified genes were validated (Figure 5) to be significantly linked to longer overall survival time (Supplementary Table S2). We insisted that these 107 genes were potential prognostic immune-related biomarkers for BRAF mutated SKCM patients.

### Protein-protein network among genes of prognostic value

**FIGURE 6:**
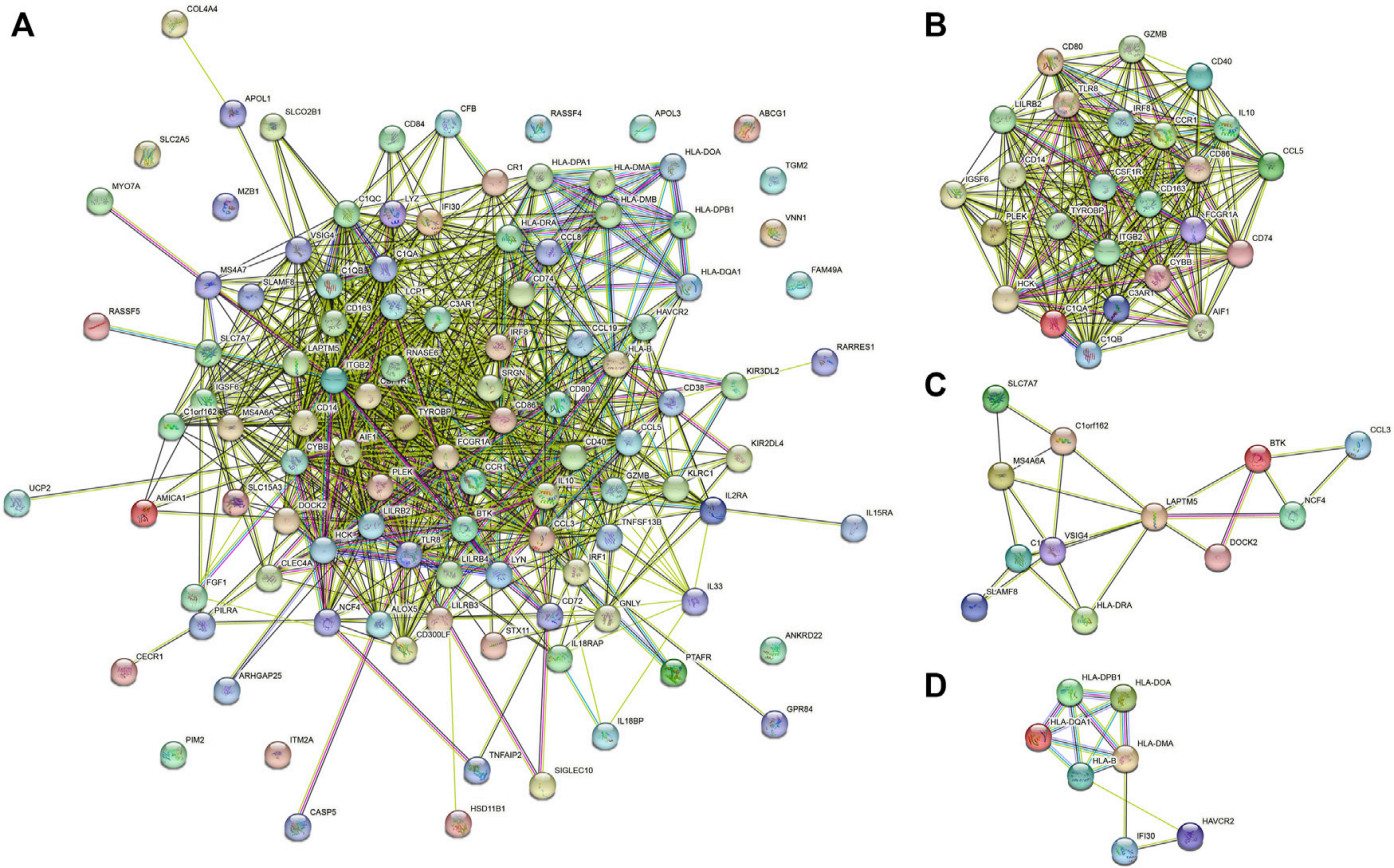
**(A)** PPI networks of 107 prognostic genes. **(B**–**D)** gene model 1, gene model 2 and gene model 3.

The protein-protein interaction (PPI) networks were constructed among 107 immune-related prognostic genes to explore their potential interactions and find the co-expression network (Figure 6A). 95 nodes and 813 edges were obtained in 107 gene interactions. Moreover, we used MCODE Plug-in to select the gene modules that interact most closely in the PPI network (module nodes> 6). In module 1 (Figure 6B), 241 edges involving 25 nodes were formed in the network. TYROBP, CD86, CSF1R, ITGB2 were found most closely related to other genes. In module 2 (12 nodes and 23 edges), LAPTM5 and VSIG4 had the higher connection values, indicating their core role in the module (Figure 6C). In module 3 (7 nodes and 13 edges), several HLA-related genes such as HLA-DQA1 and HLA-DPB1 had the higher connection values (Figure 6D).

### Selection of hub genes and their co-expression network

**FIGURE 7:**
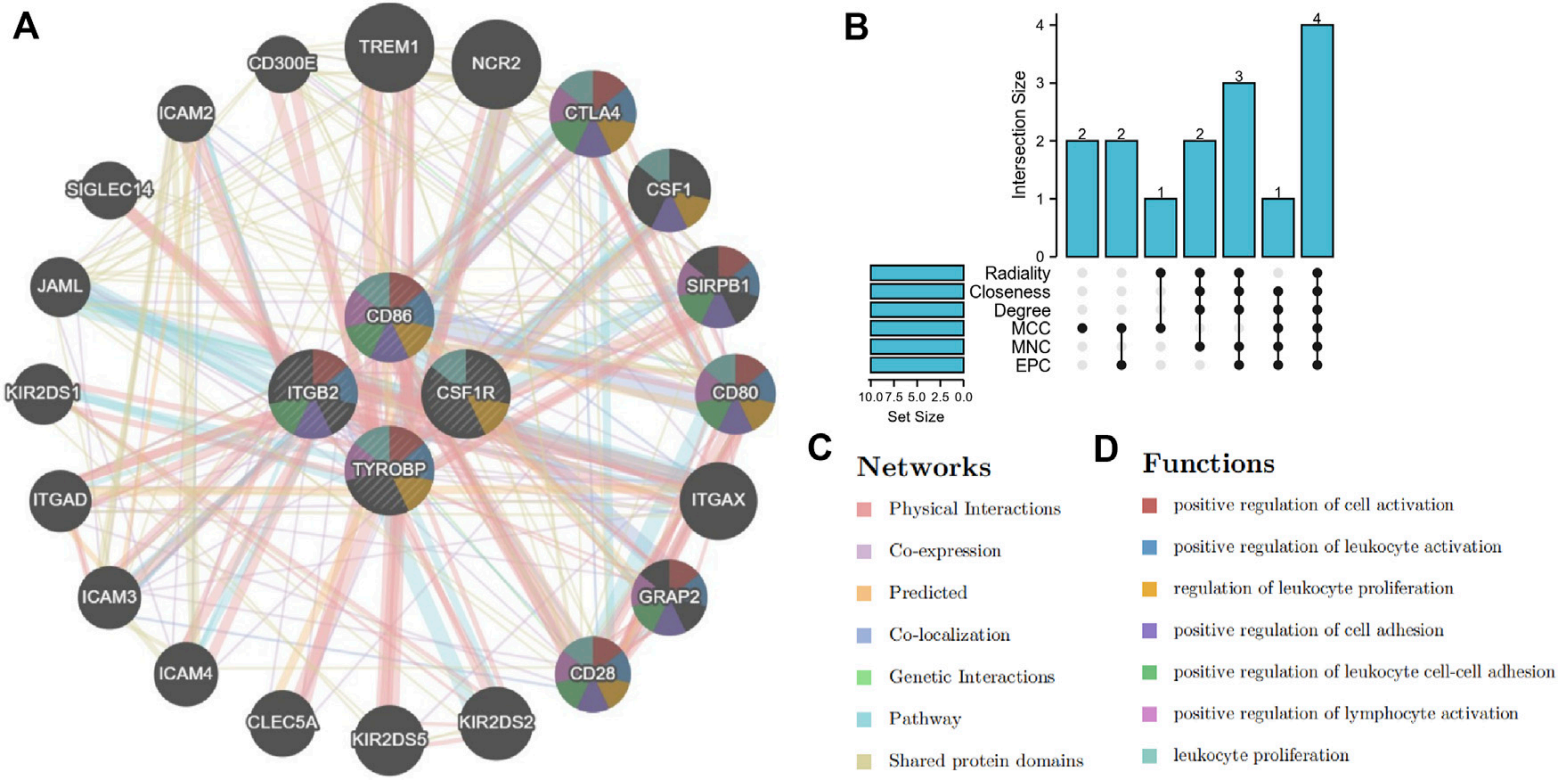
Selection and co-expression network of hub genes. **(A)** co-expression network of hub genes. **(B)** screening hub genes based on six algorithms. **(C,D)** function analysis and networks of hub genes and their co-expression genes.

Based on the above PPI network, we evaluated the top 10 genes of BRAF patients using six algorithms (Table 2). Eventually, TYROBP, CD86, CSF1R and ITGB2 were present in six algorithms at the same time (Figure 7B). By constructing the co-expression network of hub genes, the genetic interactions and pathways were analyzed (Figure 7A). Function annotation revealed that these hub genes and their co-expression genes were mainly related to leukocyte activation and lymphocyte proliferation (Figures 7C,D).

**TABLE 2:** Screening of hub genes using six algorithms in cytoHubba. The bold value represents Hub genes

Rank	EPC	MNC	MCC	Degree	Closeness	Radiality
1	**TYROBP**	**TYROBP**	**TYROBP**	**TYROBP**	**TYROBP**	**TYROBP**
2	C1QB	C1QB	C1QB	C1QB	C1QB	**CD86**
3	**CD86**	**CD86**	**CD86**	**CD86**	**CD86**	IRF8
4	IRF8	IRF8	**CSF1R**	IRF8	IRF8	CD80
5	**CSF1R**	CD80	FCGR1A	CD80	CD80	**CSF1R**
6	CD163	**CSF1R**	**ITGB2**	**CSF1R**	**CSF1R**	CD163
6	**ITGB2**	CD163	CCR1	CD163	CD163	**ITGB2**
8	CCR1	**ITGB2**	C1QA	**ITGB2**	**ITGB2**	IL10
9	LILRB2	IL10	CYBB	IL10	IL10	LILRB2
10	CYBB	LILRB2	TLR8	LILRB2	LILRB2	TLR8

### Immune infiltration results between high vs. low group and their association with hub genes

**FIGURE 8:**
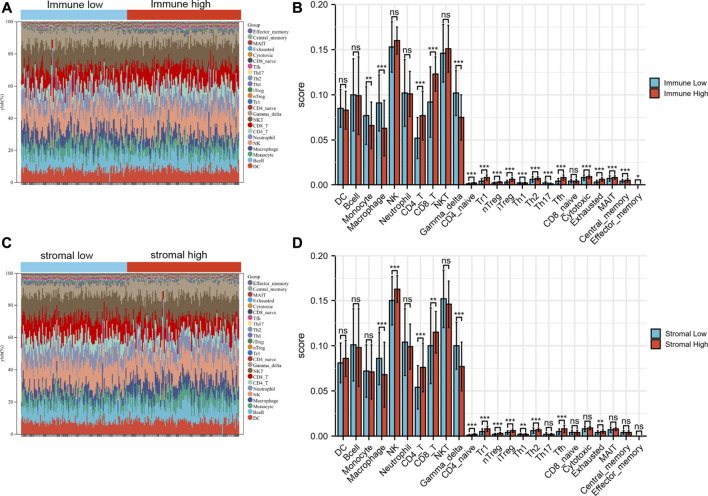
The immune landscape of BRAF mutated samples microenvironment. **(A)** The landscape of immune cells among immune high and low group. **(B)** Immune cell differences between immune high and low group. **(C)** The landscape of immune cells among stromal high and low group. **(D)** Immune cell differences between stromal high and low group. *p < 0.05; **p < 0.01; ***p < 0.001.

Based on the expression data and ImmuCellAI algorithm, we quantified the immune cell components of BRAF mutated SKCM patients (Figures 8A,C). As shown in the figure, several cells (Monocyte, Macrophage and Gamma delta cells) have been found to be significantly decreased in immune-high group. While CD4^+^T, CD8^+^T, CD4 naïve, Tr1, Th2 and many T cell subsets were significantly increased in immune-high group (Figure 8B). Similarly, when compared the stromal-high and stromal-low group, Macrophage and Gamma delta cells were found to be significantly decreased in high group. While CD4^+^T, CD8^+^T, CD4 naïve, Tr1, Th2 and many T cell subsets were significantly increased in stromal-high patients. (Figure 8D).

**FIGURE 9:**
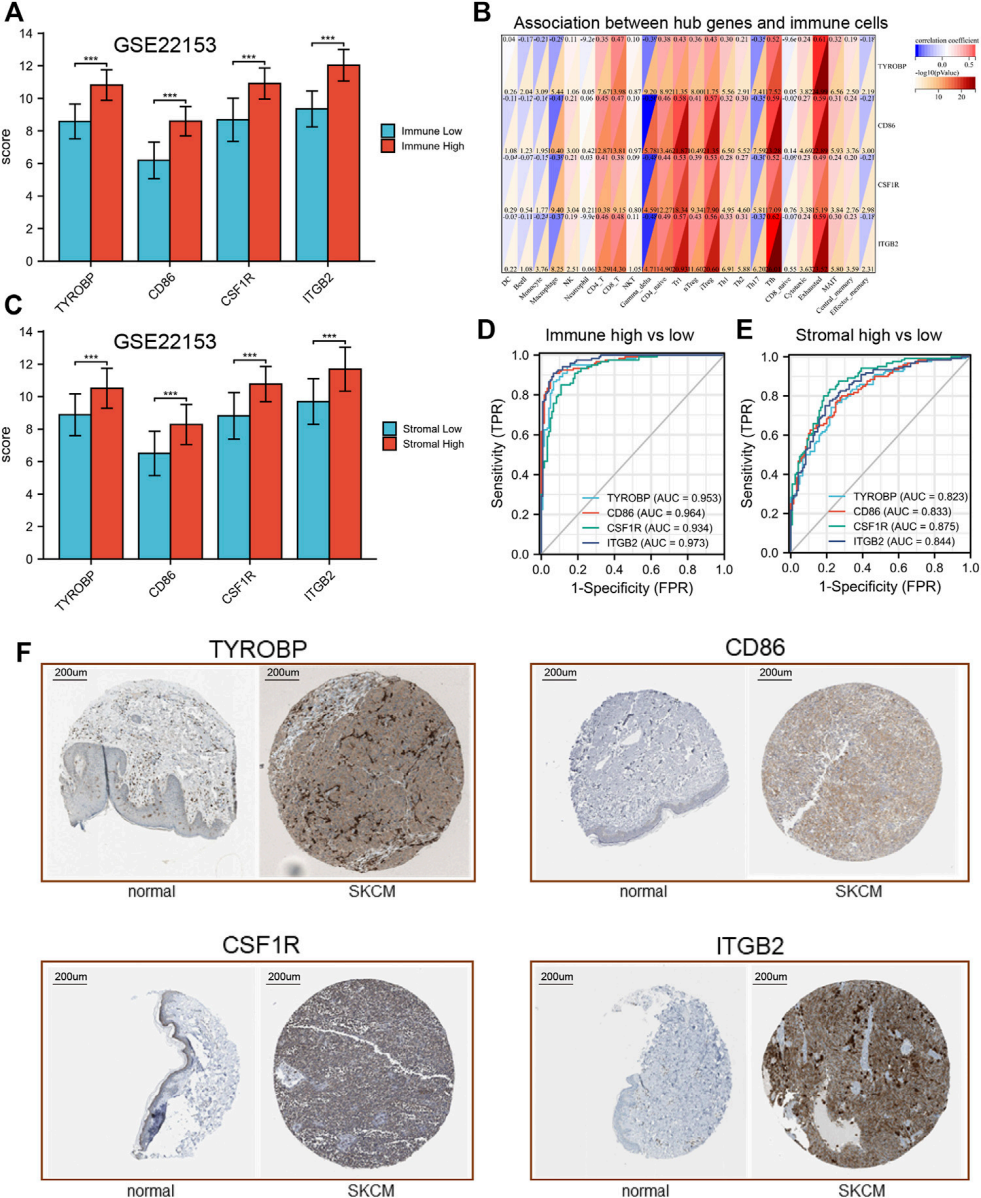
Validation of hub genes expression. **(A)** expression levels of hub genes in GSE22153. **(B)** association between hub genes and immune cells. **(C)** expression levels of hub genes in GSE22153. **(D**,**E)**, ROC curves of hub genes. **(F)** IHC results of hub genes.

Additionally, our results revealed that the expression of these hub genes may be related to the imbalance of immune cells (Figure 9B). For example, 4 hub genes (TYROBP, CD86, CSF1R and ITGB2) were mainly positively related to CD4^+^T, CD8^+^T, CD4 naïve, Tr1, iTreg, Tfh and many T cell in SKCM. While these hub genes could be found to be significantly negatively related to Macrophage and Gamma delta cells.

### Validation of hub genes expression and their ROC curves

In order to verify our results, transcriptional data of GSE22153 was used to analysis the expression of these hub genes. Our results showed that all of the hub gene expression results were consistent with the previous description (Figures 9A,C). In addition, these hub genes have good efficacy in the diagnosis of immune-high and low group (AUC > 0.82, Figure 9D). Similar results could be found between stromal high and stromal low group (Figure 9E). The IHC results indicated that these four hub genes were significantly differentially expressed between normal and tumor tissues (Figure 9F).

## Discussion

Skin cutaneous melanoma (SKCM) is one of the dead cancers with high malignant metastasis and mortality rates (Siegel et al., 2020). Identification of oncogenes provides novel insights into the progression of cancer therapy. BRAF oncogene was found in more than 50% of skin cutaneous melanoma as well as other cancers such as colorectal cancer and papillary thyroid cancer (Pollock and Meltzer, 2002; Rajagopalan et al., 2002; Cabanillas et al., 2020). The majority of researches claimed that BRAF mutation was often associated with high risk of metastasis, recurrence and poor survival outcomes (Ascierto et al., 2019b). BRAF inhibitor was considered the foundation of BRAF mutated melanoma treatment, and have demonstrated success and enhanced patient survival. However, only about 33% of patients benefit from target therapy in 5-year overall survival and the major limitations include short response duration, development of drug tolerance, and off-target effects (Robert et al., 2019). Recently, a growing group of researches showed that oncogenic BRAF can decrease the ability of the immune system to recognize melanoma cells. And the inhibition of BRAF can restore tumor immune recognition (Boni et al., 2010). Previous studies based on mice demonstrated that BRAF inhibitor response durations *in vivo* were significantly longer when melanoma cell lines were grown in immunocompetent mice compared to immunocompromised (Smalley, 2020). Besides, BRAF inhibition was associated with increased infiltration of CD4^+^T, CD8^+^T cells and reduced levels of myeloid-derived suppressor cells (MDSCs), tumor-associated macrophages (TAMs). While the depletion of CD4^+^ and CD8^+^ T cells significantly blunted the BRAF inhibitors response (Ribas et al., 2019b). Over the past years, the combination of immune therapy (anti-PD-1, anti-CTLA-4) and target therapy (BRAF inhibitor) has achieved an impressive improvement of the patients’ survival (Erkes et al., 2020). All results above suggested that the tumor microenvironment and immune effects play a vital role in SKCM therapy. However, the exploration of BRAF mutated immune microenvironment and the identification of immune-related prognostic targets in SKCM patients are still lacking (Boussadia et al., 2018).

Tumor immune microenvironment (TME) is described as significantly affecting the cancer treatment and prognosis (Hinshaw and Shevde, 2019). The ESTIMATE has been applied in glioma, renal cell carcinoma and gastrointestinal tumors, showing the validity of this algorithm in estimating tumor purity (Alonso et al., 2017; Jia et al., 2018). Therefore, ESTIMATE algorithm was applied to identify immune-related prognostic genes that contributed to patients’ overall survival by investigating the TME. Our research showed that both of the immune and stromal scores were inversely correlated with Breslow depth and AJCC stage, which have been considered as classical prognostic factors for SKCM. As shown in the Kaplan-Meier survival curve that patients with a higher immune score had longer overall survival time than those with a lower immune score in the BRAF mutated SKCM.

Through the DEGs analysis, we found that there were 990 overlapped genes which both upregulated in the immune and stromal groups. Function annotation indicated that immune response, defense response, positive regulation of immune system process and cytokine binding were important biological processes of the 990 overlapped genes. Pathway analysis demonstrated that the majority of the overlapped genes served a role in chemokine signaling pathways, cytokine-cytokine receptor interaction and cell adhesion molecules. As expected, dysregulation of immune function had a significant impact on the microenvironment of BRAF mutant SKCM patients. Among them, chemokine signaling is mainly involved in the recruitment of various immune cells, and their dysregulation may be an important reason for reducing the level of immune infiltration and leading to poor prognosis in patients with BRAF mutation. Based on our results and previous researches, it is conceivable to hypothesize that chemokines and immune response play a vital role in the regulation of SKCM TME (Huang et al., 2020; Li et al., 2020).

Subsequently, 755 genes (76%) were found correlated with longer overall survival time. This further demonstrated the clinical value of these immune microenvironment-related genes in patients with BRAF mutations. Subsequently, 107 prognostic genes of BRAF patients were verified in GEO data set. PPI network and model analysis had identified 4 hub genes (TYROBP, CD86, CSF1R and ITGB2) in our study. Moreover, our hub genes occupied a central position in both the PPI network and Model 1, proving their core position and clinical value. They were mainly associated with oncogenic transformation, immune response and regulating of immune cells (D'Angelo et al., 2019; Casey et al., 2016). For example, related studies have shown that the activation of T cells requires costimulatory signals produced by the interaction of CD28 and CD86, which could increase the infiltration of T cells in tumor tissue and prolong the survival time of mice (Jia et al., 2022). The combination of anti-PD1 and anti-CSF1 receptor (CSF1R) antibodies induced the regression of melanoma in-driven transplanted mice (Neubert et al., 2018). ITGB2 is associated with immune infiltration of multiple immune cell subsets, such as CD45, CD8, CD4T cells, CD20B cells and so on (Kwak et al., 2021). Although there was no direct evidence for the association between TYROBP and melanoma, given the important association between these hub genes and immune infiltration, we regard them as potential therapeutic targets for patients with BRAF mutations.

Previous studied demonstrated that the imbalance of immune cell components was closely related to progressive disease and poor prognosis (Qiao et al., 2019). Therefore, we conducted a further immune infiltration analysis. As expected, CD4^+^T, CD8^+^T, CD4 naïve, Tr1, Th2 and many T cell subsets were significantly increased in immune-high group. While several cells (Monocyte, Macrophage and Gamma delta cells) have been found to be significantly decreased in immune-high BRAF mutated SKCM group. There were significant differences in immune-infiltration between the two groups, which may help to identify groups that are more responsive to BRAF inhibitors. Monocyte-lymphocyte ratio (MLR) is considered to be an important indicator of tumor prognosis (Garcia et al., 2022). It has been reported that cancer-associated Macrophage play a key role in tumor progression, angiogenesis, invasion and recruitment of immunosuppressive cells (Samain and Sanz-Moreno, 2020). Persistent immune-related gene expression and T-cell penetration were associated with clinical benefit in SKCM patients (Shoushtari et al., 2022). The infiltrating levels of various effector T cells, such as CD4^+^ and CD8^+^ T, were significantly higher in the immune-high group than in the control group. After binding to MHC class I antigens on tumor cells *via* T cell receptors, CD8^+^ T cells can produce granzymes and perforin to destroy cancer cells (Tsukumo and Yasutomo, 2018). It is well known that CD8^+^ T cells have an antitumor effect, and the increase of CD8^+^ T cells can significantly improve the prognosis of SKCM patients (Chen et al., 2018). It is important to note the emerging role of CD4^+^ T cells in antitumor immunity, and in particular, their functional versatility in the context of the tumor immune microenvironment. In actual tumor therapy, the single immune function of CD8^+^ cells is not enough to destroy tumor cells, as the immune checkpoint inhibitors (anti-PD-1, anti-CTLA-4) are only 30% effective (Gellrich et al., 2020). Recent studies have found that the best initiation and maturation of MHC-I-restricted CD8^+^T cells is CTL (cytotoxic T lymphocytes), which requires the response of CD4^+^T cells (Alspach et al., 2019). By secreting interferon and promoting the proliferation and lethality of CD8^+^T cells in TME, CD4^+^T cells play a vital role (Zhu et al., 2015; Borst et al., 2018). In preclinical studies, it was found that BRAF inhibition led to increased CD40L expression and IFN-γ release from CD4^+^T cells, and decreased levels of multiple cytokines including IL1, IL6, and IL10 (Ott et al., 2013). Therefore, we speculate that increasing the proportion of CD4^+^T cells to enhance the lethality of CD8^+^T cells in TME may be a potential strategy to improve the prognosis of BRAF mutated SKCM patients.

Moreover, the expression of these hub genes was related to the imbalance of multiple immune cells. For example, 4 hub genes (TYROBP, CD86, CSF1R and ITGB2) were mainly positively related to CD4^+^T, CD8^+^T, CD4 naïve, Tr1, iTreg, Tfh and many T cell in SKCM. While these hub genes could be found to be significantly negatively related to Macrophage and Gamma delta cells. These results were consistent with their previous association with longer overall survival. There are few study on the relationship between the hub genes and BRAF mutated SKCM treatment. Therefore, we had identified several immune-related prognostic biomarkers for BRAF mutated patients. Finally, we preliminarily validated the expression of hub genes in another dataset and evaluated their diagnostic value. Our results showed that all of the hub genes significantly up-regulated in immune-high group. These data provide reference for further development of treatment for patients with BRAF mutations.

We must acknowledge the limitations in this study. First, more patients should be collected in the future to expand the sample size, which is conducive to a deeper understanding of the mechanisms of BRAF mutated SKCM and immune dysfunction. Second, we have limited experimental data and further function validation is required to investigate the interaction between the prognostic genes and immune cells.

## Conclusion

For the first time in this study, we try to explore the TME to better understand the potential prognostic immune‐related targets and mechanisms in BRAF mutated SKCM patients. This study revealed that the dysregulation of immune function and immune cells may contribute to the poor outcomes of BRAF mutated patients. It is of great significance to our further understanding of the TME and immune dysfunction in BRAF mutated SKCM.

## Data Availability

The original contributions presented in the study are included in the article/Supplementary Material, further inquiries can be directed to the corresponding author.

## Abbreviations

TME, tumor microenvironment; SKCM, skin cutaneous melanoma; GEO, Gene Expression Omnibus; TCGA, The Cancer Genome Atlas; DEGs, differentially expressed genes; PPI, protein−protein interaction; NS, normal skin; AJCC, American Joint Committee on Cancer; FDR, false discovery rate; FC, fold changes.
