# Supplementary material for: Data-driven analysis to identify prognostic immune-related biomarkers in BRAF mutated cutaneous melanoma microenvironment
Source: Front Genet. 2022 Nov 30;13:1081418. doi: 10.3389/fgene.2022.1081418 (PMC9749953; doi:10.3389/fgene.2022.1081418)
Supplement: Supplementary file 1 [file Table1.DOC]

**Supplementary Table 1. Prognostic immune-related genes in BRAF mutated SKCM patients from TCGA (P<0.01)**

|  | Gene ID | HR[exp(coef)] | coef | 95% CI lower | 95% CI upper | Z | P value |
| --- | --- | --- | --- | --- | --- | --- | --- |
| 1 | GBP4 | 0.820214 | -0.19819 | -0.25919 | -0.13719 | -6.36769 | 1.92E-10 |
| 2 | GBP2 | 0.77047 | -0.26075 | -0.34107 | -0.18044 | -6.36303 | 1.98E-10 |
| 3 | GBP1 | 0.816731 | -0.20245 | -0.26792 | -0.13697 | -6.06041 | 1.36E-09 |
| 4 | IDO1 | 0.856599 | -0.15479 | -0.20492 | -0.10465 | -6.05093 | 1.44E-09 |
| 5 | GBP5 | 0.848203 | -0.16464 | -0.21831 | -0.11096 | -6.01209 | 1.83E-09 |
| 6 | TRIM22 | 0.774827 | -0.25512 | -0.33839 | -0.17184 | -6.00426 | 1.92E-09 |
| 7 | RARRES3 | 0.813997 | -0.2058 | -0.27337 | -0.13823 | -5.9695 | 2.38E-09 |
| 8 | CXCL10 | 0.854158 | -0.15764 | -0.21008 | -0.1052 | -5.89201 | 3.82E-09 |
| 9 | CCL8 | 0.811999 | -0.20826 | -0.27759 | -0.13892 | -5.88731 | 3.93E-09 |
| 10 | CXCL11 | 0.840483 | -0.17378 | -0.23182 | -0.11574 | -5.86836 | 4.40E-09 |
| 11 | CXCL9 | 0.866643 | -0.14313 | -0.19194 | -0.09432 | -5.74757 | 9.05E-09 |
| 12 | CD80 | 0.783523 | -0.24396 | -0.32757 | -0.16034 | -5.71828 | 1.08E-08 |
| 13 | LOC400759 | 0.828371 | -0.18829 | -0.25296 | -0.12362 | -5.70664 | 1.15E-08 |
| 14 | PLA2G2D | 0.879526 | -0.12837 | -0.17272 | -0.08403 | -5.67376 | 1.40E-08 |
| 15 | GPR171 | 0.817766 | -0.20118 | -0.27083 | -0.13153 | -5.66095 | 1.51E-08 |
| 16 | CD72 | 0.785428 | -0.24153 | -0.32522 | -0.15783 | -5.65595 | 1.55E-08 |
| 17 | ZNF831 | 0.830373 | -0.18588 | -0.25034 | -0.12142 | -5.65193 | 1.59E-08 |
| 18 | P2RY13 | 0.816573 | -0.20264 | -0.27338 | -0.1319 | -5.61442 | 1.97E-08 |
| 19 | SEMA4D | 0.74133 | -0.29931 | -0.40391 | -0.19471 | -5.60847 | 2.04E-08 |
| 20 | KLRD1 | 0.825763 | -0.19145 | -0.25859 | -0.12431 | -5.58862 | 2.29E-08 |
| 21 | SP140 | 0.813532 | -0.20637 | -0.27884 | -0.1339 | -5.58135 | 2.39E-08 |
| 22 | CD38 | 0.854352 | -0.15741 | -0.21298 | -0.10185 | -5.55243 | 2.82E-08 |
| 23 | APOL3 | 0.807602 | -0.21369 | -0.28912 | -0.13825 | -5.55209 | 2.82E-08 |
| 24 | STAT4 | 0.80396 | -0.21821 | -0.29526 | -0.14115 | -5.54992 | 2.86E-08 |
| 25 | AKAP5 | 0.785144 | -0.24189 | -0.32744 | -0.15634 | -5.54168 | 3.00E-08 |
| 26 | APOBEC3G | 0.802255 | -0.22033 | -0.29854 | -0.14212 | -5.52169 | 3.36E-08 |
| 27 | SRGN | 0.751034 | -0.2863 | -0.38813 | -0.18448 | -5.51099 | 3.57E-08 |
| 28 | LAG3 | 0.846506 | -0.16664 | -0.22602 | -0.10725 | -5.49974 | 3.80E-08 |
| 29 | KLRK1 | 0.840486 | -0.17378 | -0.23576 | -0.11179 | -5.49472 | 3.91E-08 |
| 30 | GIMAP7 | 0.785188 | -0.24183 | -0.32813 | -0.15553 | -5.49229 | 3.97E-08 |
| 31 | IL18RAP | 0.779077 | -0.24965 | -0.33891 | -0.16038 | -5.48114 | 4.23E-08 |
| 32 | GCNT1 | 0.788318 | -0.23785 | -0.32314 | -0.15257 | -5.46631 | 4.59E-08 |
| 33 | CD69 | 0.831836 | -0.18412 | -0.25018 | -0.11806 | -5.46266 | 4.69E-08 |
| 34 | SAMSN1 | 0.823982 | -0.19361 | -0.26311 | -0.12411 | -5.4599 | 4.76E-08 |
| 35 | TIGIT | 0.855004 | -0.15665 | -0.21295 | -0.10035 | -5.45358 | 4.94E-08 |
| 36 | JAKMIP1 | 0.834364 | -0.18109 | -0.24637 | -0.1158 | -5.43636 | 5.44E-08 |
| 37 | PYHIN1 | 0.844991 | -0.16843 | -0.22935 | -0.1075 | -5.41846 | 6.01E-08 |
| 38 | PTPN22 | 0.823645 | -0.19402 | -0.26442 | -0.12361 | -5.40105 | 6.63E-08 |
| 39 | DPYD | 0.80967 | -0.21113 | -0.28796 | -0.1343 | -5.38586 | 7.21E-08 |
| 40 | SAMHD1 | 0.771309 | -0.25967 | -0.35428 | -0.16505 | -5.37893 | 7.49E-08 |
| 41 | CMAH | 0.77722 | -0.25203 | -0.34387 | -0.1602 | -5.37889 | 7.49E-08 |
| 42 | CXCR2P1 | 0.86439 | -0.14573 | -0.19912 | -0.09235 | -5.35023 | 8.78E-08 |
| 43 | FGL2 | 0.843078 | -0.1707 | -0.23334 | -0.10805 | -5.34079 | 9.25E-08 |
| 44 | BIRC3 | 0.834778 | -0.18059 | -0.24688 | -0.1143 | -5.33965 | 9.31E-08 |
| 45 | HLA-DRB1 | 0.814038 | -0.20575 | -0.2813 | -0.13019 | -5.33734 | 9.43E-08 |
| 46 | IRF1 | 0.786828 | -0.23975 | -0.32795 | -0.15155 | -5.32759 | 9.95E-08 |
| 47 | HLA-DPB2 | 0.790434 | -0.23517 | -0.32174 | -0.14861 | -5.32466 | 1.01E-07 |
| 48 | IL21R | 0.846864 | -0.16622 | -0.22756 | -0.10487 | -5.31086 | 1.09E-07 |
| 49 | IL2RA | 0.828053 | -0.18868 | -0.25835 | -0.11901 | -5.3077 | 1.11E-07 |
| 50 | ARHGAP25 | 0.78075 | -0.2475 | -0.33899 | -0.15602 | -5.30241 | 1.14E-07 |
| 51 | CD8B | 0.861913 | -0.1486 | -0.20356 | -0.09365 | -5.29987 | 1.16E-07 |
| 52 | RUFY4 | 0.765776 | -0.26687 | -0.36561 | -0.16812 | -5.29691 | 1.18E-07 |
| 53 | SH2D1A | 0.852021 | -0.16014 | -0.21941 | -0.10087 | -5.29568 | 1.19E-07 |
| 54 | TLR8 | 0.834306 | -0.18115 | -0.24821 | -0.1141 | -5.29467 | 1.19E-07 |
| 55 | IL15 | 0.810744 | -0.2098 | -0.28758 | -0.13202 | -5.28683 | 1.24E-07 |
| 56 | FAM105A | 0.804914 | -0.21702 | -0.29757 | -0.13647 | -5.28028 | 1.29E-07 |
| 57 | LAX1 | 0.843206 | -0.17054 | -0.23389 | -0.1072 | -5.27693 | 1.31E-07 |
| 58 | ZNF80 | 0.775616 | -0.2541 | -0.34848 | -0.15972 | -5.27675 | 1.31E-07 |
| 59 | CD1D | 0.758741 | -0.2761 | -0.37887 | -0.17332 | -5.26541 | 1.40E-07 |
| 60 | SLFN12L | 0.795221 | -0.22914 | -0.31476 | -0.14351 | -5.24505 | 1.56E-07 |
| 61 | PDCD1 | 0.857693 | -0.15351 | -0.2109 | -0.09612 | -5.24268 | 1.58E-07 |
| 62 | TNFSF13B | 0.842029 | -0.17194 | -0.23624 | -0.10764 | -5.2408 | 1.60E-07 |
| 63 | PSMB9 | 0.79592 | -0.22826 | -0.31377 | -0.14274 | -5.2316 | 1.68E-07 |
| 64 | FAM26F | 0.837701 | -0.17709 | -0.24351 | -0.11067 | -5.22579 | 1.73E-07 |
| 65 | PTPRC | 0.856372 | -0.15505 | -0.21336 | -0.09674 | -5.21153 | 1.87E-07 |
| 66 | FCRL6 | 0.805316 | -0.21652 | -0.29795 | -0.13509 | -5.21135 | 1.87E-07 |
| 67 | LILRB1 | 0.83334 | -0.18231 | -0.25112 | -0.11351 | -5.19329 | 2.07E-07 |
| 68 | NCF1B | 0.822259 | -0.1957 | -0.26965 | -0.12175 | -5.18713 | 2.14E-07 |
| 69 | DNAJC5B | 0.809708 | -0.21108 | -0.29087 | -0.13129 | -5.18521 | 2.16E-07 |
| 70 | CLECL1 | 0.787371 | -0.23906 | -0.32967 | -0.14844 | -5.17059 | 2.33E-07 |
| 71 | FCRL3 | 0.859363 | -0.15156 | -0.20907 | -0.09406 | -5.16581 | 2.39E-07 |
| 72 | TFEC | 0.833614 | -0.18199 | -0.25104 | -0.11293 | -5.1655 | 2.40E-07 |
| 73 | GPR141 | 0.762589 | -0.27104 | -0.37397 | -0.1681 | -5.16064 | 2.46E-07 |
| 74 | APOL1 | 0.828463 | -0.18818 | -0.25967 | -0.11669 | -5.15924 | 2.48E-07 |
| 75 | CXCR6 | 0.840553 | -0.17369 | -0.23979 | -0.1076 | -5.1508 | 2.59E-07 |
| 76 | SLA2 | 0.838833 | -0.17574 | -0.24271 | -0.10878 | -5.14392 | 2.69E-07 |
| 77 | C9orf139 | 0.736045 | -0.30646 | -0.42333 | -0.1896 | -5.13986 | 2.75E-07 |
| 78 | SAMD3 | 0.828012 | -0.18873 | -0.26077 | -0.11668 | -5.13434 | 2.83E-07 |
| 79 | HCG26 | 0.813439 | -0.20648 | -0.28539 | -0.12758 | -5.12902 | 2.91E-07 |
| 80 | KIR2DL4 | 0.82091 | -0.19734 | -0.2728 | -0.12188 | -5.12569 | 2.96E-07 |
| 81 | ZBP1 | 0.846624 | -0.1665 | -0.23017 | -0.10283 | -5.12536 | 2.97E-07 |
| 82 | CXCL13 | 0.888016 | -0.11877 | -0.16425 | -0.07328 | -5.11782 | 3.09E-07 |
| 83 | CD2 | 0.857555 | -0.15367 | -0.21264 | -0.0947 | -5.10732 | 3.27E-07 |
| 84 | THEMIS | 0.845245 | -0.16813 | -0.23266 | -0.1036 | -5.1064 | 3.28E-07 |
| 85 | C16orf54 | 0.827606 | -0.18922 | -0.2619 | -0.11654 | -5.10279 | 3.35E-07 |
| 86 | CD274 | 0.812027 | -0.20822 | -0.2882 | -0.12824 | -5.10262 | 3.35E-07 |
| 87 | IFNG | 0.840869 | -0.17332 | -0.23989 | -0.10675 | -5.1026 | 3.35E-07 |
| 88 | GVIN1 | 0.834076 | -0.18143 | -0.25124 | -0.11163 | -5.09411 | 3.50E-07 |
| 89 | CCL4 | 0.828672 | -0.18793 | -0.26036 | -0.1155 | -5.08542 | 3.67E-07 |
| 90 | KLRC1 | 0.790736 | -0.23479 | -0.32535 | -0.14423 | -5.08132 | 3.75E-07 |
| 91 | CLEC7A | 0.817856 | -0.20107 | -0.27869 | -0.12345 | -5.0773 | 3.83E-07 |
| 92 | HCP5 | 0.836319 | -0.17874 | -0.24776 | -0.10973 | -5.07586 | 3.86E-07 |
| 93 | SNX20 | 0.824604 | -0.19285 | -0.26733 | -0.11838 | -5.07531 | 3.87E-07 |
| 94 | CD3G | 0.851891 | -0.1603 | -0.22228 | -0.09831 | -5.06868 | 4.01E-07 |
| 95 | TRAT1 | 0.843675 | -0.16999 | -0.23589 | -0.10408 | -5.0553 | 4.30E-07 |
| 96 | MIR155HG | 0.820591 | -0.19773 | -0.27453 | -0.12094 | -5.04654 | 4.50E-07 |
| 97 | ERMN | 0.775753 | -0.25392 | -0.35267 | -0.15517 | -5.03987 | 4.66E-07 |
| 98 | HLA-DPB1 | 0.809299 | -0.21159 | -0.29393 | -0.12925 | -5.0365 | 4.74E-07 |
| 99 | CD247 | 0.843304 | -0.17043 | -0.2368 | -0.10406 | -5.03272 | 4.84E-07 |
| 100 | CD8A | 0.87091 | -0.13822 | -0.1921 | -0.08433 | -5.02723 | 4.98E-07 |
| 101 | GPR65 | 0.819434 | -0.19914 | -0.27682 | -0.12146 | -5.0246 | 5.04E-07 |
| 102 | CLEC4A | 0.779217 | -0.24947 | -0.34683 | -0.15211 | -5.02203 | 5.11E-07 |
| 103 | TNFRSF9 | 0.849554 | -0.16304 | -0.22669 | -0.0994 | -5.02074 | 5.15E-07 |
| 104 | TBX21 | 0.83661 | -0.1784 | -0.2481 | -0.1087 | -5.01635 | 5.27E-07 |
| 105 | RHOH | 0.846879 | -0.1662 | -0.2312 | -0.10119 | -5.01097 | 5.42E-07 |
| 106 | RASGRP1 | 0.834434 | -0.181 | -0.25189 | -0.11012 | -5.00454 | 5.60E-07 |
| 107 | CCR2 | 0.840444 | -0.17382 | -0.24191 | -0.10574 | -5.00409 | 5.61E-07 |
| 108 | GIMAP4 | 0.7916 | -0.2337 | -0.32535 | -0.14205 | -4.99788 | 5.80E-07 |
| 109 | RSAD2 | 0.829209 | -0.18728 | -0.26081 | -0.11376 | -4.99263 | 5.96E-07 |
| 110 | GZMK | 0.869455 | -0.13989 | -0.19488 | -0.0849 | -4.98591 | 6.17E-07 |
| 111 | CRTAM | 0.835628 | -0.17957 | -0.25016 | -0.10898 | -4.98575 | 6.17E-07 |
| 112 | TARP | 0.821759 | -0.19631 | -0.2735 | -0.11912 | -4.98474 | 6.20E-07 |
| 113 | ABCD2 | 0.819112 | -0.19953 | -0.27806 | -0.12101 | -4.9805 | 6.34E-07 |
| 114 | KCNA3 | 0.830314 | -0.18595 | -0.25918 | -0.11272 | -4.97689 | 6.46E-07 |
| 115 | MS4A6A | 0.807609 | -0.21368 | -0.2979 | -0.12946 | -4.97278 | 6.60E-07 |
| 116 | SLAMF6 | 0.859751 | -0.15111 | -0.21068 | -0.09155 | -4.97222 | 6.62E-07 |
| 117 | HLA-DOA | 0.85337 | -0.15856 | -0.22108 | -0.09605 | -4.9712 | 6.65E-07 |
| 118 | CYBB | 0.834921 | -0.18042 | -0.25157 | -0.10927 | -4.97007 | 6.69E-07 |
| 119 | IPCEF1 | 0.822235 | -0.19573 | -0.273 | -0.11846 | -4.9645 | 6.89E-07 |
| 120 | PDCD1LG2 | 0.819936 | -0.19853 | -0.27699 | -0.12007 | -4.95938 | 7.07E-07 |
| 121 | HLA-B | 0.811908 | -0.20837 | -0.29076 | -0.12598 | -4.95691 | 7.16E-07 |
| 122 | HLA-DMB | 0.819112 | -0.19953 | -0.27846 | -0.12061 | -4.95481 | 7.24E-07 |
| 123 | CXorf21 | 0.806828 | -0.21465 | -0.2996 | -0.12969 | -4.95194 | 7.35E-07 |
| 124 | GZMA | 0.861893 | -0.14862 | -0.20745 | -0.08979 | -4.95147 | 7.37E-07 |
| 125 | CIITA | 0.858812 | -0.15221 | -0.21246 | -0.09196 | -4.95135 | 7.37E-07 |
| 126 | MNDA | 0.829174 | -0.18732 | -0.26148 | -0.11317 | -4.95108 | 7.38E-07 |
| 127 | CD86 | 0.813392 | -0.20654 | -0.28832 | -0.12477 | -4.95022 | 7.41E-07 |
| 128 | RGS18 | 0.819677 | -0.19885 | -0.27762 | -0.12007 | -4.9473 | 7.53E-07 |
| 129 | TRAF3IP3 | 0.826387 | -0.19069 | -0.26629 | -0.11509 | -4.94369 | 7.67E-07 |
| 130 | C1QA | 0.825465 | -0.19181 | -0.26786 | -0.11576 | -4.94314 | 7.69E-07 |
| 131 | TTC24 | 0.799117 | -0.22425 | -0.31326 | -0.13524 | -4.93779 | 7.90E-07 |
| 132 | HLA-DRA | 0.853574 | -0.15832 | -0.22123 | -0.09542 | -4.93286 | 8.10E-07 |
| 133 | P2RY10 | 0.848847 | -0.16388 | -0.22914 | -0.09861 | -4.92113 | 8.60E-07 |
| 134 | CLIC2 | 0.815992 | -0.20335 | -0.28447 | -0.12223 | -4.91328 | 8.96E-07 |
| 135 | FAM46C | 0.830977 | -0.18515 | -0.25905 | -0.11125 | -4.91065 | 9.08E-07 |
| 136 | TRANK1 | 0.792169 | -0.23298 | -0.32606 | -0.1399 | -4.90563 | 9.31E-07 |
| 137 | KIAA0748 | 0.84629 | -0.16689 | -0.23379 | -0.1 | -4.88999 | 1.01E-06 |
| 138 | SPATC1 | 0.692134 | -0.36798 | -0.51556 | -0.22039 | -4.88691 | 1.02E-06 |
| 139 | HLA-DMA | 0.800427 | -0.22261 | -0.31195 | -0.13327 | -4.88392 | 1.04E-06 |
| 140 | CCR5 | 0.845454 | -0.16788 | -0.23529 | -0.10048 | -4.88165 | 1.05E-06 |
| 141 | CD3D | 0.871627 | -0.13739 | -0.19258 | -0.08221 | -4.87997 | 1.06E-06 |
| 142 | FASLG | 0.850521 | -0.16191 | -0.22698 | -0.09683 | -4.87635 | 1.08E-06 |
| 143 | HLA-DPA1 | 0.833347 | -0.18231 | -0.25575 | -0.10886 | -4.86521 | 1.14E-06 |
| 144 | GIMAP5 | 0.789409 | -0.23647 | -0.3318 | -0.14114 | -4.86162 | 1.16E-06 |
| 145 | UBD | 0.886012 | -0.12102 | -0.16995 | -0.0721 | -4.84819 | 1.25E-06 |
| 146 | CD74 | 0.833094 | -0.18261 | -0.25645 | -0.10877 | -4.84693 | 1.25E-06 |
| 147 | PTGER2 | 0.801353 | -0.22145 | -0.31106 | -0.13185 | -4.84378 | 1.27E-06 |
| 148 | PARP15 | 0.826208 | -0.19091 | -0.26819 | -0.11363 | -4.84185 | 1.29E-06 |
| 149 | DTHD1 | 0.804638 | -0.21736 | -0.30545 | -0.12928 | -4.83661 | 1.32E-06 |
| 150 | TIMD4 | 0.821033 | -0.19719 | -0.27712 | -0.11726 | -4.83553 | 1.33E-06 |
| 151 | CD226 | 0.816722 | -0.20246 | -0.28454 | -0.12038 | -4.83434 | 1.34E-06 |
| 152 | MIAT | 0.778939 | -0.24982 | -0.35115 | -0.1485 | -4.83241 | 1.35E-06 |
| 153 | HSH2D | 0.843383 | -0.17033 | -0.23943 | -0.10124 | -4.83166 | 1.35E-06 |
| 154 | HCLS1 | 0.804951 | -0.21697 | -0.30502 | -0.12893 | -4.82992 | 1.37E-06 |
| 155 | ATP8B4 | 0.808311 | -0.21281 | -0.29921 | -0.12641 | -4.82772 | 1.38E-06 |
| 156 | EPSTI1 | 0.848759 | -0.16398 | -0.23056 | -0.0974 | -4.82735 | 1.38E-06 |
| 157 | HLA-DOB | 0.849901 | -0.16264 | -0.22876 | -0.09651 | -4.82039 | 1.43E-06 |
| 158 | CD200R1 | 0.811225 | -0.20921 | -0.29428 | -0.12414 | -4.82021 | 1.43E-06 |
| 159 | EVI2B | 0.828013 | -0.18873 | -0.26563 | -0.11182 | -4.80999 | 1.51E-06 |
| 160 | TMSL3 | 0.774635 | -0.25536 | -0.35944 | -0.15128 | -4.80883 | 1.52E-06 |
| 161 | CCDC88B | 0.771096 | -0.25994 | -0.36592 | -0.15396 | -4.80733 | 1.53E-06 |
| 162 | GPR114 | 0.848471 | -0.16432 | -0.23133 | -0.09731 | -4.806 | 1.54E-06 |
| 163 | BCL2L14 | 0.785821 | -0.24103 | -0.33939 | -0.14267 | -4.8028 | 1.56E-06 |
| 164 | TLR2 | 0.800837 | -0.2221 | -0.3129 | -0.1313 | -4.79426 | 1.63E-06 |
| 165 | C2orf85 | 0.767915 | -0.26408 | -0.37203 | -0.15612 | -4.79425 | 1.63E-06 |
| 166 | TBC1D10C | 0.849903 | -0.16263 | -0.2292 | -0.09606 | -4.78817 | 1.68E-06 |
| 167 | PSTPIP1 | 0.843661 | -0.17 | -0.23973 | -0.10028 | -4.77889 | 1.76E-06 |
| 168 | 1-Mar | 0.801941 | -0.22072 | -0.31127 | -0.13017 | -4.77737 | 1.78E-06 |
| 169 | PRF1 | 0.85627 | -0.15517 | -0.21886 | -0.09147 | -4.77473 | 1.80E-06 |
| 170 | BTLA | 0.84167 | -0.17237 | -0.24315 | -0.10159 | -4.77286 | 1.82E-06 |
| 171 | XCL2 | 0.842764 | -0.17107 | -0.24134 | -0.1008 | -4.77149 | 1.83E-06 |
| 172 | FCGR3A | 0.839517 | -0.17493 | -0.2468 | -0.10306 | -4.77025 | 1.84E-06 |
| 173 | TMEM156 | 0.836126 | -0.17898 | -0.25256 | -0.10539 | -4.76726 | 1.87E-06 |
| 174 | LILRB2 | 0.836651 | -0.17835 | -0.25174 | -0.10495 | -4.76277 | 1.91E-06 |
| 175 | SIRPG | 0.873431 | -0.13533 | -0.19102 | -0.07963 | -4.76194 | 1.92E-06 |
| 176 | SIT1 | 0.860735 | -0.14997 | -0.21177 | -0.08816 | -4.75586 | 1.98E-06 |
| 177 | GIMAP1 | 0.788082 | -0.23815 | -0.3363 | -0.14 | -4.75577 | 1.98E-06 |
| 178 | SLA | 0.829636 | -0.18677 | -0.26377 | -0.10976 | -4.7538 | 2.00E-06 |
| 179 | ABCB1 | 0.822716 | -0.19514 | -0.27575 | -0.11454 | -4.74511 | 2.08E-06 |
| 180 | NLRC4 | 0.763231 | -0.27019 | -0.3819 | -0.15849 | -4.7409 | 2.13E-06 |
| 181 | FLT3LG | 0.774736 | -0.25523 | -0.36081 | -0.14966 | -4.73819 | 2.16E-06 |
| 182 | GAB3 | 0.775103 | -0.25476 | -0.36029 | -0.14923 | -4.73169 | 2.23E-06 |
| 183 | C8orf80 | 0.835575 | -0.17963 | -0.25407 | -0.1052 | -4.72974 | 2.25E-06 |
| 184 | FYB | 0.830878 | -0.18527 | -0.26207 | -0.10848 | -4.72861 | 2.26E-06 |
| 185 | CD27 | 0.860782 | -0.14991 | -0.21206 | -0.08777 | -4.72818 | 2.27E-06 |
| 186 | ARHGAP9 | 0.830585 | -0.18563 | -0.26258 | -0.10867 | -4.72774 | 2.27E-06 |
| 187 | PILRA | 0.786206 | -0.24054 | -0.3405 | -0.14058 | -4.71631 | 2.40E-06 |
| 188 | HLA-DQB1 | 0.845943 | -0.1673 | -0.23698 | -0.09763 | -4.70641 | 2.52E-06 |
| 189 | C1QC | 0.836121 | -0.17898 | -0.25363 | -0.10433 | -4.69944 | 2.61E-06 |
| 190 | VNN2 | 0.838609 | -0.17601 | -0.24948 | -0.10254 | -4.69572 | 2.66E-06 |
| 191 | PLEK | 0.837371 | -0.17749 | -0.25164 | -0.10334 | -4.69127 | 2.72E-06 |
| 192 | IGJ | 0.905257 | -0.09954 | -0.14116 | -0.05792 | -4.68734 | 2.77E-06 |
| 193 | TAGAP | 0.831556 | -0.18446 | -0.2617 | -0.10721 | -4.68045 | 2.86E-06 |
| 194 | LCP2 | 0.817085 | -0.20201 | -0.28662 | -0.1174 | -4.6795 | 2.88E-06 |
| 195 | CCR8 | 0.793839 | -0.23088 | -0.3276 | -0.13415 | -4.67833 | 2.89E-06 |
| 196 | CFB | 0.839531 | -0.17491 | -0.24822 | -0.1016 | -4.67638 | 2.92E-06 |
| 197 | NCF1 | 0.861399 | -0.1492 | -0.21179 | -0.0866 | -4.67169 | 2.99E-06 |
| 198 | UBASH3A | 0.86037 | -0.15039 | -0.21349 | -0.08729 | -4.67128 | 2.99E-06 |
| 199 | ANKRD22 | 0.869805 | -0.13949 | -0.19805 | -0.08092 | -4.66817 | 3.04E-06 |
| 200 | RLTPR | 0.854978 | -0.15668 | -0.22259 | -0.09077 | -4.65905 | 3.18E-06 |
| 201 | ADAM28 | 0.856717 | -0.15465 | -0.21972 | -0.08958 | -4.65808 | 3.19E-06 |
| 202 | C1QB | 0.850211 | -0.16227 | -0.23057 | -0.09397 | -4.65659 | 3.21E-06 |
| 203 | LRMP | 0.834862 | -0.18049 | -0.25664 | -0.10434 | -4.64563 | 3.39E-06 |
| 204 | ADORA2A | 0.765186 | -0.26764 | -0.38064 | -0.15463 | -4.6418 | 3.45E-06 |
| 205 | NLRC3 | 0.788295 | -0.23788 | -0.33836 | -0.13741 | -4.64047 | 3.48E-06 |
| 206 | GZMH | 0.864733 | -0.14533 | -0.20673 | -0.08394 | -4.6396 | 3.49E-06 |
| 207 | NKG7 | 0.873077 | -0.13573 | -0.19312 | -0.07834 | -4.63565 | 3.56E-06 |
| 208 | CTSW | 0.870341 | -0.13887 | -0.19759 | -0.08015 | -4.63551 | 3.56E-06 |
| 209 | P2RY14 | 0.789217 | -0.23671 | -0.33689 | -0.13654 | -4.63129 | 3.63E-06 |
| 210 | AMICA1 | 0.830665 | -0.18553 | -0.26405 | -0.107 | -4.63074 | 3.64E-06 |
| 211 | CD7 | 0.872256 | -0.13667 | -0.19459 | -0.07876 | -4.6252 | 3.74E-06 |
| 212 | IL15RA | 0.798242 | -0.22534 | -0.32092 | -0.12977 | -4.62132 | 3.81E-06 |
| 213 | CXCR3 | 0.867937 | -0.14164 | -0.20177 | -0.0815 | -4.61605 | 3.91E-06 |
| 214 | PTPRCAP | 0.849674 | -0.1629 | -0.23207 | -0.09373 | -4.61601 | 3.91E-06 |
| 215 | C17orf87 | 0.826747 | -0.19026 | -0.27107 | -0.10944 | -4.61432 | 3.94E-06 |
| 216 | CSF1 | 0.782705 | -0.245 | -0.34912 | -0.14088 | -4.61205 | 3.99E-06 |
| 217 | KLRC4 | 0.809612 | -0.2112 | -0.30098 | -0.12142 | -4.61088 | 4.01E-06 |
| 218 | IL4I1 | 0.829496 | -0.18694 | -0.26649 | -0.10739 | -4.60578 | 4.11E-06 |
| 219 | ZC3H12D | 0.80692 | -0.21453 | -0.30585 | -0.12322 | -4.60461 | 4.13E-06 |
| 220 | ZBTB32 | 0.801651 | -0.22108 | -0.31519 | -0.12697 | -4.60421 | 4.14E-06 |
| 221 | HCST | 0.819472 | -0.19909 | -0.28387 | -0.11432 | -4.60318 | 4.16E-06 |
| 222 | AOAH | 0.866486 | -0.14331 | -0.20441 | -0.08221 | -4.59726 | 4.28E-06 |
| 223 | GPR174 | 0.839419 | -0.17505 | -0.24969 | -0.1004 | -4.5962 | 4.30E-06 |
| 224 | STAC3 | 0.777074 | -0.25222 | -0.35985 | -0.14459 | -4.59291 | 4.37E-06 |
| 225 | CYSLTR1 | 0.79564 | -0.22861 | -0.32632 | -0.13089 | -4.5854 | 4.53E-06 |
| 226 | ADAMDEC1 | 0.888776 | -0.11791 | -0.16839 | -0.06744 | -4.57851 | 4.68E-06 |
| 227 | ARRDC5 | 0.746658 | -0.29215 | -0.41733 | -0.16697 | -4.57428 | 4.78E-06 |
| 228 | IL12RB1 | 0.85618 | -0.15528 | -0.22184 | -0.08871 | -4.57179 | 4.84E-06 |
| 229 | KLHDC7B | 0.847182 | -0.16584 | -0.23694 | -0.09474 | -4.57138 | 4.85E-06 |
| 230 | FGD2 | 0.833898 | -0.18164 | -0.25956 | -0.10373 | -4.56924 | 4.89E-06 |
| 231 | SLFN11 | 0.815503 | -0.20395 | -0.29151 | -0.11639 | -4.56521 | 4.99E-06 |
| 232 | ZBED2 | 0.865796 | -0.14411 | -0.206 | -0.08221 | -4.56314 | 5.04E-06 |
| 233 | KIR3DL2 | 0.75325 | -0.28336 | -0.40516 | -0.16156 | -4.55974 | 5.12E-06 |
| 234 | CD96 | 0.866379 | -0.14343 | -0.20511 | -0.08175 | -4.55765 | 5.17E-06 |
| 235 | IL18BP | 0.781482 | -0.24656 | -0.35271 | -0.14042 | -4.55282 | 5.29E-06 |
| 236 | HLA-DRB5 | 0.852729 | -0.15931 | -0.22805 | -0.09058 | -4.54274 | 5.55E-06 |
| 237 | 1-Sep | 0.821352 | -0.1968 | -0.28177 | -0.11184 | -4.53973 | 5.63E-06 |
| 238 | ACSL5 | 0.827021 | -0.18993 | -0.27197 | -0.10789 | -4.53736 | 5.70E-06 |
| 239 | FCGR2C | 0.828597 | -0.18802 | -0.2693 | -0.10675 | -4.53418 | 5.78E-06 |
| 240 | PIK3CG | 0.847088 | -0.16595 | -0.23785 | -0.09405 | -4.52362 | 6.08E-06 |
| 241 | AIF1 | 0.823709 | -0.19394 | -0.27806 | -0.10982 | -4.51856 | 6.23E-06 |
| 242 | ACAP1 | 0.863918 | -0.14628 | -0.20981 | -0.08274 | -4.51262 | 6.40E-06 |
| 243 | CD48 | 0.853618 | -0.15827 | -0.22701 | -0.08953 | -4.51247 | 6.41E-06 |
| 244 | GRIN3A | 0.763999 | -0.26919 | -0.38617 | -0.15221 | -4.51025 | 6.48E-06 |
| 245 | GIMAP6 | 0.795555 | -0.22871 | -0.32812 | -0.12931 | -4.50968 | 6.49E-06 |
| 246 | CCL5 | 0.864953 | -0.14508 | -0.20815 | -0.08201 | -4.50846 | 6.53E-06 |
| 247 | SLAMF1 | 0.873961 | -0.13472 | -0.1933 | -0.07614 | -4.50717 | 6.57E-06 |
| 248 | IKZF1 | 0.851232 | -0.16107 | -0.23117 | -0.09097 | -4.5032 | 6.69E-06 |
| 249 | P2RY12 | 0.836898 | -0.17805 | -0.25566 | -0.10045 | -4.49694 | 6.89E-06 |
| 250 | ITGAL | 0.868132 | -0.14141 | -0.20306 | -0.07976 | -4.49583 | 6.93E-06 |
| 251 | LOC96610 | 0.882266 | -0.12526 | -0.17992 | -0.0706 | -4.49165 | 7.07E-06 |
| 252 | CASS4 | 0.813117 | -0.20688 | -0.29718 | -0.11658 | -4.49018 | 7.12E-06 |
| 253 | ZMYND15 | 0.807023 | -0.2144 | -0.30801 | -0.1208 | -4.48933 | 7.14E-06 |
| 254 | ICOS | 0.859546 | -0.15135 | -0.2175 | -0.0852 | -4.4842 | 7.32E-06 |
| 255 | C11orf21 | 0.822846 | -0.19499 | -0.28024 | -0.10973 | -4.48269 | 7.37E-06 |
| 256 | CD3E | 0.874287 | -0.13435 | -0.1931 | -0.07559 | -4.48174 | 7.40E-06 |
| 257 | HLA-DQA1 | 0.864311 | -0.14582 | -0.2096 | -0.08205 | -4.48137 | 7.42E-06 |
| 258 | IKZF3 | 0.864222 | -0.14593 | -0.20978 | -0.08207 | -4.47882 | 7.51E-06 |
| 259 | LCK | 0.8757 | -0.13273 | -0.19096 | -0.0745 | -4.46738 | 7.92E-06 |
| 260 | CLEC4E | 0.861182 | -0.14945 | -0.21513 | -0.08377 | -4.45971 | 8.21E-06 |
| 261 | EMB | 0.849541 | -0.16306 | -0.2348 | -0.09132 | -4.45461 | 8.40E-06 |
| 262 | FCRL5 | 0.894751 | -0.11121 | -0.16017 | -0.06225 | -4.45175 | 8.52E-06 |
| 263 | LST1 | 0.827562 | -0.18927 | -0.27262 | -0.10592 | -4.45053 | 8.57E-06 |
| 264 | SECTM1 | 0.839044 | -0.17549 | -0.25279 | -0.0982 | -4.44994 | 8.59E-06 |
| 265 | SLC27A2 | 0.833495 | -0.18213 | -0.26246 | -0.10179 | -4.44341 | 8.85E-06 |
| 266 | CLEC9A | 0.804788 | -0.21718 | -0.313 | -0.12136 | -4.44233 | 8.90E-06 |
| 267 | KIAA0125 | 0.878408 | -0.12964 | -0.18686 | -0.07243 | -4.44134 | 8.94E-06 |
| 268 | TLR10 | 0.844604 | -0.16889 | -0.24355 | -0.09423 | -4.43361 | 9.27E-06 |
| 269 | RASAL3 | 0.849167 | -0.1635 | -0.23583 | -0.09117 | -4.43041 | 9.41E-06 |
| 270 | ACRBP | 0.778735 | -0.25008 | -0.36076 | -0.13941 | -4.42866 | 9.48E-06 |
| 271 | APBB1IP | 0.845716 | -0.16757 | -0.24177 | -0.09337 | -4.42638 | 9.58E-06 |
| 272 | GPR82 | 0.791887 | -0.23334 | -0.33677 | -0.1299 | -4.42145 | 9.80E-06 |
| 273 | CECR1 | 0.83474 | -0.18063 | -0.26075 | -0.10052 | -4.41924 | 9.91E-06 |
| 274 | LOC100240735 | 0.782824 | -0.24485 | -0.35344 | -0.13625 | -4.41901 | 9.92E-06 |
| 275 | HK3 | 0.842368 | -0.17154 | -0.24768 | -0.0954 | -4.4155 | 1.01E-05 |
| 276 | TSHR | 0.782001 | -0.2459 | -0.35509 | -0.1367 | -4.41368 | 1.02E-05 |
| 277 | NCF1C | 0.863578 | -0.14667 | -0.21189 | -0.08145 | -4.40771 | 1.04E-05 |
| 278 | CP | 0.878403 | -0.12965 | -0.18743 | -0.07187 | -4.39783 | 1.09E-05 |
| 279 | LOC606724 | 0.822295 | -0.19566 | -0.28291 | -0.1084 | -4.39473 | 1.11E-05 |
| 280 | SPOCK2 | 0.834584 | -0.18082 | -0.26149 | -0.10015 | -4.39333 | 1.12E-05 |
| 281 | S1PR4 | 0.84495 | -0.16848 | -0.24375 | -0.09321 | -4.38687 | 1.15E-05 |
| 282 | EBI3 | 0.851372 | -0.16091 | -0.23282 | -0.08899 | -4.38553 | 1.16E-05 |
| 283 | CTLA4 | 0.865406 | -0.14456 | -0.20917 | -0.07994 | -4.38466 | 1.16E-05 |
| 284 | LOC100233209 | 0.840079 | -0.17426 | -0.2522 | -0.09632 | -4.38227 | 1.17E-05 |
| 285 | PTPN7 | 0.858824 | -0.15219 | -0.2203 | -0.08409 | -4.37981 | 1.19E-05 |
| 286 | RASSF4 | 0.788117 | -0.23811 | -0.34472 | -0.1315 | -4.37762 | 1.20E-05 |
| 287 | GZMB | 0.878279 | -0.12979 | -0.18795 | -0.07163 | -4.37413 | 1.22E-05 |
| 288 | BCAS4 | 0.778707 | -0.25012 | -0.36223 | -0.13801 | -4.37288 | 1.23E-05 |
| 289 | PIM2 | 0.769574 | -0.26192 | -0.37932 | -0.14452 | -4.37257 | 1.23E-05 |
| 290 | CORO1A | 0.851695 | -0.16053 | -0.23275 | -0.08831 | -4.35643 | 1.32E-05 |
| 291 | LIME1 | 0.820139 | -0.19828 | -0.28751 | -0.10905 | -4.3552 | 1.33E-05 |
| 292 | C10orf128 | 0.809311 | -0.21157 | -0.30692 | -0.11623 | -4.3493 | 1.37E-05 |
| 293 | SIGLEC10 | 0.862891 | -0.14747 | -0.21395 | -0.08099 | -4.34764 | 1.38E-05 |
| 294 | AIM2 | 0.888862 | -0.11781 | -0.17093 | -0.06469 | -4.34707 | 1.38E-05 |
| 295 | CYTIP | 0.861281 | -0.14933 | -0.21678 | -0.08189 | -4.33984 | 1.43E-05 |
| 296 | PLAC8 | 0.857631 | -0.15358 | -0.22295 | -0.08422 | -4.33958 | 1.43E-05 |
| 297 | CTSS | 0.822516 | -0.19539 | -0.28376 | -0.10702 | -4.3335 | 1.47E-05 |
| 298 | BIN2 | 0.853943 | -0.15789 | -0.22931 | -0.08648 | -4.33328 | 1.47E-05 |
| 299 | IGSF6 | 0.834096 | -0.18141 | -0.26347 | -0.09934 | -4.33255 | 1.47E-05 |
| 300 | TSPAN32 | 0.833945 | -0.18159 | -0.26375 | -0.09943 | -4.33192 | 1.48E-05 |
| 301 | IL2RG | 0.870848 | -0.13829 | -0.2009 | -0.07567 | -4.32854 | 1.50E-05 |
| 302 | SLC8A1 | 0.819248 | -0.19937 | -0.28971 | -0.10903 | -4.32535 | 1.52E-05 |
| 303 | LAT2 | 0.815092 | -0.20445 | -0.29715 | -0.11176 | -4.323 | 1.54E-05 |
| 304 | FCGR1A | 0.849753 | -0.16281 | -0.23665 | -0.08897 | -4.32139 | 1.55E-05 |
| 305 | CCR1 | 0.822493 | -0.19542 | -0.28422 | -0.10661 | -4.31289 | 1.61E-05 |
| 306 | TNFAIP8 | 0.787576 | -0.2388 | -0.34739 | -0.13021 | -4.31005 | 1.63E-05 |
| 307 | CARD11 | 0.867511 | -0.14213 | -0.2068 | -0.07745 | -4.30697 | 1.66E-05 |
| 308 | ARHGAP15 | 0.829624 | -0.18678 | -0.27187 | -0.1017 | -4.30254 | 1.69E-05 |
| 309 | CYTH4 | 0.820767 | -0.19752 | -0.28754 | -0.10749 | -4.30033 | 1.71E-05 |
| 310 | MGC29506 | 0.911337 | -0.09284 | -0.13524 | -0.05045 | -4.29203 | 1.77E-05 |
| 311 | MYO1F | 0.818495 | -0.20029 | -0.29179 | -0.10878 | -4.2901 | 1.79E-05 |
| 312 | SIRPB2 | 0.829792 | -0.18658 | -0.27192 | -0.10124 | -4.28495 | 1.83E-05 |
| 313 | HLA-DQB2 | 0.881903 | -0.12567 | -0.18319 | -0.06816 | -4.28276 | 1.85E-05 |
| 314 | HSD11B1 | 0.85935 | -0.15158 | -0.22096 | -0.0822 | -4.28221 | 1.85E-05 |
| 315 | NCF4 | 0.819199 | -0.19943 | -0.29072 | -0.10813 | -4.28147 | 1.86E-05 |
| 316 | NCKAP1L | 0.853605 | -0.15829 | -0.23078 | -0.0858 | -4.27975 | 1.87E-05 |
| 317 | C5orf20 | 0.864969 | -0.14506 | -0.21159 | -0.07854 | -4.2739 | 1.92E-05 |
| 318 | HAVCR2 | 0.835501 | -0.17972 | -0.26216 | -0.09729 | -4.27326 | 1.93E-05 |
| 319 | TNFRSF18 | 0.847389 | -0.1656 | -0.24167 | -0.08952 | -4.26628 | 1.99E-05 |
| 320 | ST8SIA4 | 0.822241 | -0.19572 | -0.28568 | -0.10577 | -4.26436 | 2.00E-05 |
| 321 | SELL | 0.859922 | -0.15091 | -0.22028 | -0.08154 | -4.26384 | 2.01E-05 |
| 322 | CD53 | 0.857474 | -0.15376 | -0.2245 | -0.08303 | -4.2607 | 2.04E-05 |
| 323 | TMEM150B | 0.840485 | -0.17378 | -0.2538 | -0.09375 | -4.25591 | 2.08E-05 |
| 324 | P2RY6 | 0.815954 | -0.2034 | -0.29715 | -0.10965 | -4.25226 | 2.12E-05 |
| 325 | TNFRSF17 | 0.880988 | -0.12671 | -0.18512 | -0.0683 | -4.25196 | 2.12E-05 |
| 326 | RGL4 | 0.819154 | -0.19948 | -0.29157 | -0.10739 | -4.24558 | 2.18E-05 |
| 327 | SUSD3 | 0.841364 | -0.17273 | -0.25248 | -0.09298 | -4.24506 | 2.19E-05 |
| 328 | GIMAP8 | 0.797029 | -0.22686 | -0.33179 | -0.12193 | -4.23758 | 2.26E-05 |
| 329 | ETV7 | 0.871533 | -0.1375 | -0.20114 | -0.07386 | -4.23464 | 2.29E-05 |
| 330 | PTK2B | 0.747512 | -0.29101 | -0.4258 | -0.15621 | -4.23137 | 2.32E-05 |
| 331 | VAV1 | 0.863813 | -0.1464 | -0.21421 | -0.07858 | -4.23118 | 2.32E-05 |
| 332 | CD40 | 0.819106 | -0.19954 | -0.292 | -0.10708 | -4.22977 | 2.34E-05 |
| 333 | CEACAM4 | 0.769776 | -0.26166 | -0.38303 | -0.14029 | -4.22542 | 2.38E-05 |
| 334 | HLA-DQA2 | 0.888226 | -0.11853 | -0.17353 | -0.06353 | -4.22388 | 2.40E-05 |
| 335 | PVRIG | 0.801234 | -0.2216 | -0.32443 | -0.11877 | -4.2238 | 2.40E-05 |
| 336 | TNFSF10 | 0.847411 | -0.16557 | -0.24255 | -0.08859 | -4.2156 | 2.49E-05 |
| 337 | PNOC | 0.860181 | -0.15061 | -0.22077 | -0.08046 | -4.20783 | 2.58E-05 |
| 338 | TNIP3 | 0.837778 | -0.177 | -0.25947 | -0.09454 | -4.20689 | 2.59E-05 |
| 339 | ARL11 | 0.809422 | -0.21143 | -0.30995 | -0.11292 | -4.2066 | 2.59E-05 |
| 340 | IL10RA | 0.85332 | -0.15862 | -0.23253 | -0.08471 | -4.20614 | 2.60E-05 |
| 341 | PIPOX | 0.770721 | -0.26043 | -0.38187 | -0.13899 | -4.20307 | 2.63E-05 |
| 342 | PRKCQ | 0.863142 | -0.14718 | -0.21586 | -0.07849 | -4.19968 | 2.67E-05 |
| 343 | TIFAB | 0.855553 | -0.15601 | -0.22888 | -0.08313 | -4.19583 | 2.72E-05 |
| 344 | C3AR1 | 0.828905 | -0.18765 | -0.27533 | -0.09996 | -4.19439 | 2.74E-05 |
| 345 | IRF8 | 0.85648 | -0.15492 | -0.22733 | -0.08252 | -4.19394 | 2.74E-05 |
| 346 | ALOX5 | 0.848529 | -0.16425 | -0.24102 | -0.08748 | -4.19322 | 2.75E-05 |
| 347 | FAM179A | 0.820139 | -0.19828 | -0.29099 | -0.10557 | -4.19185 | 2.77E-05 |
| 348 | GNGT2 | 0.816047 | -0.20328 | -0.29838 | -0.10819 | -4.1899 | 2.79E-05 |
| 349 | KIAA0040 | 0.823133 | -0.19464 | -0.28584 | -0.10344 | -4.18299 | 2.88E-05 |
| 350 | TNFRSF4 | 0.842926 | -0.17088 | -0.25097 | -0.09078 | -4.18125 | 2.90E-05 |
| 351 | DAPP1 | 0.858961 | -0.15203 | -0.22331 | -0.08076 | -4.18063 | 2.91E-05 |
| 352 | GGTA1 | 0.833442 | -0.18219 | -0.26765 | -0.09673 | -4.17829 | 2.94E-05 |
| 353 | ECEL1 | 0.831004 | -0.18512 | -0.27206 | -0.09818 | -4.17312 | 3.00E-05 |
| 354 | COL4A3 | 0.83317 | -0.18252 | -0.26824 | -0.0968 | -4.1731 | 3.00E-05 |
| 355 | CD33 | 0.832732 | -0.18304 | -0.26902 | -0.09707 | -4.17285 | 3.01E-05 |
| 356 | CD300LF | 0.842904 | -0.1709 | -0.25124 | -0.09056 | -4.16932 | 3.06E-05 |
| 357 | FLT3 | 0.837596 | -0.17722 | -0.26058 | -0.09386 | -4.16686 | 3.09E-05 |
| 358 | CD6 | 0.871979 | -0.13699 | -0.20145 | -0.07253 | -4.16542 | 3.11E-05 |
| 359 | LTA | 0.84912 | -0.16355 | -0.24053 | -0.08658 | -4.16475 | 3.12E-05 |
| 360 | INPP5D | 0.836202 | -0.17889 | -0.26315 | -0.09462 | -4.16087 | 3.17E-05 |
| 361 | ZNF683 | 0.861503 | -0.14908 | -0.21943 | -0.07872 | -4.15319 | 3.28E-05 |
| 362 | TLR9 | 0.819116 | -0.19953 | -0.29377 | -0.10529 | -4.1497 | 3.33E-05 |
| 363 | CCL4L2 | 0.856156 | -0.1553 | -0.22868 | -0.08193 | -4.14841 | 3.35E-05 |
| 364 | XCL1 | 0.850148 | -0.16234 | -0.23905 | -0.08564 | -4.14822 | 3.35E-05 |
| 365 | DOCK8 | 0.847334 | -0.16566 | -0.24398 | -0.08734 | -4.14567 | 3.39E-05 |
| 366 | CLEC10A | 0.873517 | -0.13523 | -0.19919 | -0.07127 | -4.14398 | 3.41E-05 |
| 367 | PTGER4 | 0.830197 | -0.18609 | -0.27413 | -0.09806 | -4.14311 | 3.43E-05 |
| 368 | SUCNR1 | 0.817694 | -0.20127 | -0.29657 | -0.10597 | -4.13932 | 3.48E-05 |
| 369 | RNASE6 | 0.809542 | -0.21129 | -0.31134 | -0.11124 | -4.13905 | 3.49E-05 |
| 370 | FAM113B | 0.853963 | -0.15787 | -0.23273 | -0.083 | -4.13292 | 3.58E-05 |
| 371 | PIK3AP1 | 0.82666 | -0.19036 | -0.28064 | -0.10008 | -4.13282 | 3.58E-05 |
| 372 | CD4 | 0.826334 | -0.19076 | -0.28125 | -0.10027 | -4.1317 | 3.60E-05 |
| 373 | CEACAM21 | 0.843006 | -0.17078 | -0.2518 | -0.08976 | -4.13125 | 3.61E-05 |
| 374 | RCSD1 | 0.834691 | -0.18069 | -0.26645 | -0.09494 | -4.12965 | 3.63E-05 |
| 375 | LGALS9 | 0.814568 | -0.2051 | -0.30245 | -0.10774 | -4.12904 | 3.64E-05 |
| 376 | CD5 | 0.879483 | -0.12842 | -0.18943 | -0.06741 | -4.1254 | 3.70E-05 |
| 377 | FCGR1C | 0.842874 | -0.17094 | -0.25222 | -0.08966 | -4.12204 | 3.76E-05 |
| 378 | PARVG | 0.845568 | -0.16775 | -0.24751 | -0.08798 | -4.12195 | 3.76E-05 |
| 379 | LY75 | 0.868042 | -0.14152 | -0.20883 | -0.0742 | -4.12043 | 3.78E-05 |
| 380 | STAP1 | 0.856526 | -0.15487 | -0.22856 | -0.08118 | -4.11893 | 3.81E-05 |
| 381 | SERPING1 | 0.818178 | -0.20068 | -0.29625 | -0.1051 | -4.1154 | 3.87E-05 |
| 382 | SLC7A7 | 0.810094 | -0.21061 | -0.31093 | -0.11028 | -4.11435 | 3.88E-05 |
| 383 | WAS | 0.845595 | -0.16772 | -0.24767 | -0.08776 | -4.11113 | 3.94E-05 |
| 384 | PTGDR | 0.789036 | -0.23694 | -0.35003 | -0.12386 | -4.10673 | 4.01E-05 |
| 385 | PTPN6 | 0.846524 | -0.16662 | -0.24628 | -0.08695 | -4.09909 | 4.15E-05 |
| 386 | GPR18 | 0.849564 | -0.16303 | -0.24101 | -0.08506 | -4.09801 | 4.17E-05 |
| 387 | FGF1 | 0.866035 | -0.14383 | -0.21282 | -0.07484 | -4.08601 | 4.39E-05 |
| 388 | FLJ40330 | 0.862031 | -0.14846 | -0.21984 | -0.07708 | -4.07659 | 4.57E-05 |
| 389 | TLR7 | 0.856641 | -0.15474 | -0.22922 | -0.08025 | -4.0716 | 4.67E-05 |
| 390 | DOK2 | 0.834737 | -0.18064 | -0.2676 | -0.09368 | -4.07142 | 4.67E-05 |
| 391 | ITGB2 | 0.847757 | -0.16516 | -0.24474 | -0.08558 | -4.06791 | 4.74E-05 |
| 392 | AMPD1 | 0.811215 | -0.20922 | -0.31003 | -0.10841 | -4.06779 | 4.75E-05 |
| 393 | FCRL1 | 0.864348 | -0.14578 | -0.21611 | -0.07545 | -4.06245 | 4.86E-05 |
| 394 | SPN | 0.865256 | -0.14473 | -0.21458 | -0.07488 | -4.06103 | 4.89E-05 |
| 395 | PIK3R6 | 0.82609 | -0.19105 | -0.28327 | -0.09883 | -4.06038 | 4.90E-05 |
| 396 | PSD4 | 0.837694 | -0.1771 | -0.2626 | -0.0916 | -4.05991 | 4.91E-05 |
| 397 | LOC647121 | 0.829356 | -0.18711 | -0.27756 | -0.09665 | -4.0541 | 5.03E-05 |
| 398 | LCP1 | 0.847899 | -0.16499 | -0.24492 | -0.08506 | -4.04572 | 5.22E-05 |
| 399 | SASH3 | 0.857862 | -0.15331 | -0.22765 | -0.07898 | -4.04235 | 5.29E-05 |
| 400 | LYZ | 0.877786 | -0.13035 | -0.19356 | -0.06714 | -4.04191 | 5.30E-05 |
| 401 | DOCK2 | 0.858668 | -0.15237 | -0.22635 | -0.0784 | -4.03712 | 5.41E-05 |
| 402 | FCGR1B | 0.851401 | -0.16087 | -0.23902 | -0.08273 | -4.03482 | 5.46E-05 |
| 403 | TNFAIP8L2 | 0.826706 | -0.19031 | -0.28279 | -0.09782 | -4.03291 | 5.51E-05 |
| 404 | SOCS1 | 0.829808 | -0.18656 | -0.27724 | -0.09588 | -4.03233 | 5.52E-05 |
| 405 | CASP5 | 0.792552 | -0.2325 | -0.34552 | -0.11948 | -4.03188 | 5.53E-05 |
| 406 | LAMP3 | 0.85963 | -0.15125 | -0.22491 | -0.0776 | -4.02471 | 5.70E-05 |
| 407 | GLIPR1 | 0.83236 | -0.18349 | -0.27289 | -0.09409 | -4.02272 | 5.75E-05 |
| 408 | ARHGAP30 | 0.840648 | -0.17358 | -0.25817 | -0.08899 | -4.02196 | 5.77E-05 |
| 409 | LILRB4 | 0.872791 | -0.13606 | -0.20244 | -0.06967 | -4.01709 | 5.89E-05 |
| 410 | SIGLEC1 | 0.846654 | -0.16646 | -0.24768 | -0.08524 | -4.01698 | 5.89E-05 |
| 411 | ASCL2 | 0.834906 | -0.18044 | -0.2685 | -0.09237 | -4.0158 | 5.92E-05 |
| 412 | MS4A1 | 0.907763 | -0.09677 | -0.14401 | -0.04953 | -4.01493 | 5.95E-05 |
| 413 | JSRP1 | 0.872103 | -0.13685 | -0.20375 | -0.06994 | -4.00904 | 6.10E-05 |
| 414 | CD84 | 0.860637 | -0.15008 | -0.22356 | -0.0766 | -4.0033 | 6.25E-05 |
| 415 | LGALS2 | 0.871793 | -0.1372 | -0.20439 | -0.07002 | -4.00276 | 6.26E-05 |
| 416 | GPR132 | 0.835559 | -0.17965 | -0.26771 | -0.0916 | -3.99889 | 6.36E-05 |
| 417 | EMR1 | 0.859717 | -0.15115 | -0.22525 | -0.07706 | -3.99842 | 6.38E-05 |
| 418 | CD300C | 0.832372 | -0.18348 | -0.27344 | -0.09351 | -3.99706 | 6.41E-05 |
| 419 | LAPTM5 | 0.844796 | -0.16866 | -0.25137 | -0.08595 | -3.99651 | 6.43E-05 |
| 420 | CCND2 | 0.836444 | -0.1786 | -0.26619 | -0.091 | -3.99629 | 6.43E-05 |
| 421 | DENND3 | 0.78853 | -0.23758 | -0.35418 | -0.12099 | -3.9939 | 6.50E-05 |
| 422 | HLA-DRB6 | 0.899096 | -0.10637 | -0.1586 | -0.05413 | -3.99116 | 6.58E-05 |
| 423 | C4A | 0.855727 | -0.1558 | -0.23248 | -0.07913 | -3.98287 | 6.81E-05 |
| 424 | FCRL2 | 0.86991 | -0.13937 | -0.20795 | -0.07078 | -3.98276 | 6.81E-05 |
| 425 | TLR4 | 0.8391 | -0.17543 | -0.26183 | -0.08902 | -3.97938 | 6.91E-05 |
| 426 | CST7 | 0.879824 | -0.12803 | -0.19112 | -0.06495 | -3.97765 | 6.96E-05 |
| 427 | GMFG | 0.824059 | -0.19351 | -0.28889 | -0.09814 | -3.9766 | 6.99E-05 |
| 428 | FCN1 | 0.870342 | -0.13887 | -0.20734 | -0.0704 | -3.97507 | 7.04E-05 |
| 429 | ARHGDIB | 0.81071 | -0.20984 | -0.3136 | -0.10609 | -3.96387 | 7.37E-05 |
| 430 | SIGLEC11 | 0.852373 | -0.15973 | -0.23876 | -0.0807 | -3.96143 | 7.45E-05 |
| 431 | BANK1 | 0.882977 | -0.12446 | -0.18611 | -0.06281 | -3.95675 | 7.60E-05 |
| 432 | C1orf162 | 0.821881 | -0.19616 | -0.29341 | -0.09891 | -3.95342 | 7.70E-05 |
| 433 | LOC100272216 | 0.806338 | -0.21525 | -0.32216 | -0.10834 | -3.94617 | 7.94E-05 |
| 434 | MS4A4A | 0.834809 | -0.18055 | -0.27035 | -0.09076 | -3.94088 | 8.12E-05 |
| 435 | CSF2RB | 0.859323 | -0.15161 | -0.22708 | -0.07613 | -3.93707 | 8.25E-05 |
| 436 | TNFRSF1B | 0.820552 | -0.19778 | -0.29625 | -0.0993 | -3.93647 | 8.27E-05 |
| 437 | RASSF5 | 0.834237 | -0.18124 | -0.27158 | -0.09089 | -3.93176 | 8.43E-05 |
| 438 | PRDM1 | 0.826033 | -0.19112 | -0.28658 | -0.09566 | -3.92402 | 8.71E-05 |
| 439 | SSTR2 | 0.777176 | -0.25209 | -0.37801 | -0.12617 | -3.92368 | 8.72E-05 |
| 440 | SELPLG | 0.840476 | -0.17379 | -0.26071 | -0.08686 | -3.91854 | 8.91E-05 |
| 441 | EVI2A | 0.854961 | -0.1567 | -0.23511 | -0.07829 | -3.91697 | 8.97E-05 |
| 442 | C2 | 0.865412 | -0.14455 | -0.21688 | -0.07222 | -3.91677 | 8.97E-05 |
| 443 | LY9 | 0.879972 | -0.12787 | -0.19187 | -0.06386 | -3.91528 | 9.03E-05 |
| 444 | LAIR1 | 0.852186 | -0.15995 | -0.24012 | -0.07978 | -3.91042 | 9.21E-05 |
| 445 | GPR84 | 0.857662 | -0.15355 | -0.23054 | -0.07655 | -3.90847 | 9.29E-05 |
| 446 | SEL1L3 | 0.870586 | -0.13859 | -0.2081 | -0.06907 | -3.90754 | 9.32E-05 |
| 447 | SH2D1B | 0.840432 | -0.17384 | -0.26109 | -0.08659 | -3.90506 | 9.42E-05 |
| 448 | APOBEC3H | 0.839404 | -0.17506 | -0.26295 | -0.08718 | -3.90426 | 9.45E-05 |
| 449 | BLNK | 0.868915 | -0.14051 | -0.21106 | -0.06996 | -3.90339 | 9.49E-05 |
| 450 | LILRA6 | 0.834899 | -0.18045 | -0.27114 | -0.08975 | -3.89952 | 9.64E-05 |
| 451 | FUT7 | 0.847408 | -0.16557 | -0.24902 | -0.08213 | -3.8889 | 1.01E-04 |
| 452 | LTB | 0.887549 | -0.11929 | -0.17948 | -0.05911 | -3.88479 | 1.02E-04 |
| 453 | CD79A | 0.909171 | -0.09522 | -0.14328 | -0.04716 | -3.88311 | 1.03E-04 |
| 454 | RAB37 | 0.862344 | -0.1481 | -0.22288 | -0.07332 | -3.88164 | 1.04E-04 |
| 455 | RTN1 | 0.869921 | -0.13935 | -0.20981 | -0.0689 | -3.87666 | 1.06E-04 |
| 456 | VCAM1 | 0.865257 | -0.14473 | -0.21811 | -0.07134 | -3.86543 | 1.11E-04 |
| 457 | P2RY8 | 0.839479 | -0.17497 | -0.26373 | -0.08621 | -3.86368 | 1.12E-04 |
| 458 | ADAM6 | 0.926732 | -0.07609 | -0.1147 | -0.03748 | -3.86288 | 1.12E-04 |
| 459 | NEXN | 0.828761 | -0.18782 | -0.28316 | -0.09249 | -3.86143 | 1.13E-04 |
| 460 | TNF | 0.843355 | -0.17037 | -0.25696 | -0.08378 | -3.85635 | 1.15E-04 |
| 461 | CSF1R | 0.856901 | -0.15443 | -0.23305 | -0.07582 | -3.85023 | 1.18E-04 |
| 462 | IL2RB | 0.869365 | -0.13999 | -0.21137 | -0.06862 | -3.84429 | 1.21E-04 |
| 463 | PLD4 | 0.876745 | -0.13154 | -0.19862 | -0.06445 | -3.84309 | 1.21E-04 |
| 464 | FPR2 | 0.847782 | -0.16513 | -0.24935 | -0.08091 | -3.84297 | 1.22E-04 |
| 465 | POU2AF1 | 0.911778 | -0.09236 | -0.13947 | -0.04525 | -3.84221 | 1.22E-04 |
| 466 | DHRS3 | 0.824749 | -0.19268 | -0.29097 | -0.09438 | -3.84174 | 1.22E-04 |
| 467 | TMPRSS3 | 0.854939 | -0.15672 | -0.23679 | -0.07666 | -3.83638 | 1.25E-04 |
| 468 | SPI1 | 0.843347 | -0.17038 | -0.25762 | -0.08313 | -3.82742 | 1.29E-04 |
| 469 | NFAM1 | 0.845331 | -0.16803 | -0.25408 | -0.08197 | -3.82683 | 1.30E-04 |
| 470 | SIGLEC5 | 0.850327 | -0.16213 | -0.24532 | -0.07894 | -3.81989 | 1.34E-04 |
| 471 | C14orf73 | 0.860605 | -0.15012 | -0.22716 | -0.07308 | -3.81898 | 1.34E-04 |
| 472 | WDFY4 | 0.875034 | -0.13349 | -0.2021 | -0.06488 | -3.81332 | 1.37E-04 |
| 473 | APOL4 | 0.865447 | -0.14451 | -0.21911 | -0.0699 | -3.79644 | 1.47E-04 |
| 474 | C6orf97 | 0.829649 | -0.18675 | -0.28324 | -0.09027 | -3.79362 | 1.48E-04 |
| 475 | LILRA1 | 0.833016 | -0.1827 | -0.2772 | -0.0882 | -3.78932 | 1.51E-04 |
| 476 | TOX | 0.882133 | -0.12541 | -0.19031 | -0.06051 | -3.78748 | 1.52E-04 |
| 477 | IL10 | 0.841956 | -0.17203 | -0.26109 | -0.08297 | -3.78589 | 1.53E-04 |
| 478 | PTPRE | 0.838665 | -0.17594 | -0.26703 | -0.08485 | -3.78573 | 1.53E-04 |
| 479 | ITK | 0.886024 | -0.12101 | -0.18367 | -0.05835 | -3.78524 | 1.54E-04 |
| 480 | IL32 | 0.868939 | -0.14048 | -0.21351 | -0.06746 | -3.77051 | 1.63E-04 |
| 481 | XIRP1 | 0.877096 | -0.13114 | -0.19938 | -0.0629 | -3.76662 | 1.65E-04 |
| 482 | IL33 | 0.878243 | -0.12983 | -0.19743 | -0.06224 | -3.76448 | 1.67E-04 |
| 483 | MAN1A1 | 0.851733 | -0.16048 | -0.24416 | -0.07681 | -3.75905 | 1.71E-04 |
| 484 | MEI1 | 0.869472 | -0.13987 | -0.2128 | -0.06693 | -3.75869 | 1.71E-04 |
| 485 | NLRP1 | 0.841761 | -0.17226 | -0.26222 | -0.0823 | -3.75288 | 1.75E-04 |
| 486 | TREML2 | 0.843888 | -0.16974 | -0.25841 | -0.08106 | -3.75171 | 1.76E-04 |
| 487 | GPR34 | 0.850752 | -0.16163 | -0.24624 | -0.07702 | -3.74423 | 1.81E-04 |
| 488 | CD19 | 0.907617 | -0.09693 | -0.14771 | -0.04615 | -3.74121 | 1.83E-04 |
| 489 | CR1 | 0.876528 | -0.13179 | -0.20087 | -0.0627 | -3.73878 | 1.85E-04 |
| 490 | GHRL | 0.790815 | -0.23469 | -0.3578 | -0.11158 | -3.73646 | 1.87E-04 |
| 491 | NCR3 | 0.853831 | -0.15802 | -0.24099 | -0.07505 | -3.73295 | 1.89E-04 |
| 492 | FERMT3 | 0.850332 | -0.16213 | -0.24736 | -0.0769 | -3.72849 | 1.93E-04 |
| 493 | KCNMA1 | 0.872893 | -0.13594 | -0.20744 | -0.06445 | -3.72669 | 1.94E-04 |
| 494 | MYO7A | 0.845644 | -0.16766 | -0.25587 | -0.07944 | -3.72492 | 1.95E-04 |
| 495 | NOD2 | 0.85508 | -0.15656 | -0.23898 | -0.07414 | -3.72316 | 1.97E-04 |
| 496 | LRRC25 | 0.828491 | -0.18815 | -0.2875 | -0.0888 | -3.71173 | 2.06E-04 |
| 497 | GRAP2 | 0.858595 | -0.15246 | -0.23306 | -0.07185 | -3.70719 | 2.10E-04 |
| 498 | IL18 | 0.872577 | -0.1363 | -0.20842 | -0.06419 | -3.70448 | 2.12E-04 |
| 499 | GFI1 | 0.860697 | -0.15001 | -0.22942 | -0.07061 | -3.70277 | 2.13E-04 |
| 500 | PLCL2 | 0.837325 | -0.17754 | -0.27154 | -0.08355 | -3.70211 | 2.14E-04 |
| 501 | POU2F2 | 0.860217 | -0.15057 | -0.23044 | -0.0707 | -3.69474 | 2.20E-04 |
| 502 | TBXAS1 | 0.845024 | -0.16839 | -0.25776 | -0.07902 | -3.69298 | 2.22E-04 |
| 503 | SIGLEC14 | 0.884258 | -0.12301 | -0.18831 | -0.0577 | -3.69158 | 2.23E-04 |
| 504 | C17orf60 | 0.842075 | -0.17189 | -0.26324 | -0.08054 | -3.68792 | 2.26E-04 |
| 505 | DEF6 | 0.835659 | -0.17953 | -0.27502 | -0.08405 | -3.68527 | 2.28E-04 |
| 506 | FCER1G | 0.863568 | -0.14668 | -0.22471 | -0.06866 | -3.68451 | 2.29E-04 |
| 507 | MYO1G | 0.862735 | -0.14765 | -0.22627 | -0.06903 | -3.68074 | 2.33E-04 |
| 508 | ZAP70 | 0.889196 | -0.11744 | -0.18004 | -0.05484 | -3.67701 | 2.36E-04 |
| 509 | MARCO | 0.909711 | -0.09463 | -0.14513 | -0.04413 | -3.67258 | 2.40E-04 |
| 510 | TMEM176B | 0.849898 | -0.16264 | -0.24944 | -0.07584 | -3.67256 | 2.40E-04 |
| 511 | DENND1C | 0.855491 | -0.15608 | -0.23959 | -0.07257 | -3.6633 | 2.49E-04 |
| 512 | CD28 | 0.846255 | -0.16694 | -0.25634 | -0.07753 | -3.65978 | 2.52E-04 |
| 513 | SLAMF8 | 0.866836 | -0.14291 | -0.21955 | -0.06627 | -3.65464 | 2.58E-04 |
| 514 | LMO2 | 0.788837 | -0.2372 | -0.36455 | -0.10984 | -3.65038 | 2.62E-04 |
| 515 | SLC12A3 | 0.814007 | -0.20579 | -0.31632 | -0.09525 | -3.649 | 2.63E-04 |
| 516 | LAT | 0.85319 | -0.15877 | -0.24411 | -0.07343 | -3.64647 | 2.66E-04 |
| 517 | DBH | 0.816826 | -0.20233 | -0.31112 | -0.09354 | -3.64505 | 2.67E-04 |
| 518 | MS4A7 | 0.84303 | -0.17075 | -0.2626 | -0.07891 | -3.64374 | 2.69E-04 |
| 519 | RNASET2 | 0.776673 | -0.25274 | -0.38898 | -0.11649 | -3.63578 | 2.77E-04 |
| 520 | CD14 | 0.847932 | -0.16495 | -0.25397 | -0.07594 | -3.63185 | 2.81E-04 |
| 521 | GPBAR1 | 0.821834 | -0.19622 | -0.3023 | -0.09013 | -3.6252 | 2.89E-04 |
| 522 | CCL2 | 0.849186 | -0.16348 | -0.25189 | -0.07506 | -3.62384 | 2.90E-04 |
| 523 | SLC43A2 | 0.808974 | -0.21199 | -0.32673 | -0.09725 | -3.62122 | 2.93E-04 |
| 524 | CCL13 | 0.874195 | -0.13445 | -0.20728 | -0.06162 | -3.61825 | 2.97E-04 |
| 525 | GNLY | 0.892853 | -0.11333 | -0.17475 | -0.05191 | -3.61657 | 2.99E-04 |
| 526 | GPR183 | 0.857376 | -0.15388 | -0.2373 | -0.07046 | -3.61529 | 3.00E-04 |
| 527 | C19orf38 | 0.825836 | -0.19136 | -0.29522 | -0.0875 | -3.61125 | 3.05E-04 |
| 528 | CCR6 | 0.848428 | -0.16437 | -0.25364 | -0.0751 | -3.60879 | 3.08E-04 |
| 529 | KLRB1 | 0.880132 | -0.12768 | -0.19711 | -0.05826 | -3.60479 | 3.12E-04 |
| 530 | KIF19 | 0.829664 | -0.18673 | -0.28827 | -0.0852 | -3.60446 | 3.13E-04 |
| 531 | TNFRSF25 | 0.851951 | -0.16023 | -0.24748 | -0.07298 | -3.59931 | 3.19E-04 |
| 532 | TNFSF14 | 0.827238 | -0.18966 | -0.29302 | -0.08631 | -3.59665 | 3.22E-04 |
| 533 | PRDM8 | 0.855574 | -0.15598 | -0.24108 | -0.07088 | -3.59254 | 3.27E-04 |
| 534 | MSR1 | 0.858376 | -0.15271 | -0.23607 | -0.06935 | -3.59062 | 3.30E-04 |
| 535 | FAM159A | 0.84565 | -0.16765 | -0.25922 | -0.07607 | -3.58818 | 3.33E-04 |
| 536 | MPEG1 | 0.865124 | -0.14488 | -0.22403 | -0.06574 | -3.58792 | 3.33E-04 |
| 537 | SLC1A3 | 0.864621 | -0.14546 | -0.22505 | -0.06588 | -3.58227 | 3.41E-04 |
| 538 | IL7R | 0.89318 | -0.11297 | -0.17478 | -0.05116 | -3.58221 | 3.41E-04 |
| 539 | CLEC12A | 0.879254 | -0.12868 | -0.19918 | -0.05818 | -3.57738 | 3.47E-04 |
| 540 | FLI1 | 0.823415 | -0.19429 | -0.3009 | -0.08769 | -3.57202 | 3.54E-04 |
| 541 | C10orf54 | 0.825571 | -0.19168 | -0.29689 | -0.08647 | -3.57077 | 3.56E-04 |
| 542 | ABI3 | 0.842883 | -0.17093 | -0.26537 | -0.07648 | -3.54719 | 3.89E-04 |
| 543 | ITGB7 | 0.861923 | -0.14859 | -0.23085 | -0.06633 | -3.54026 | 4.00E-04 |
| 544 | MRC1 | 0.876268 | -0.13208 | -0.20524 | -0.05893 | -3.53864 | 4.02E-04 |
| 545 | PLCB2 | 0.840619 | -0.17362 | -0.27004 | -0.07719 | -3.52889 | 4.17E-04 |
| 546 | CD37 | 0.86297 | -0.14738 | -0.22933 | -0.06542 | -3.52435 | 4.25E-04 |
| 547 | SYTL3 | 0.838844 | -0.17573 | -0.27357 | -0.07789 | -3.52017 | 4.31E-04 |
| 548 | AGAP2 | 0.829582 | -0.18683 | -0.29089 | -0.08278 | -3.51908 | 4.33E-04 |
| 549 | PRAM1 | 0.858145 | -0.15298 | -0.23843 | -0.06754 | -3.5092 | 4.49E-04 |
| 550 | DPEP2 | 0.847501 | -0.16546 | -0.25798 | -0.07295 | -3.50542 | 4.56E-04 |
| 551 | ALOX5AP | 0.8755 | -0.13296 | -0.20738 | -0.05855 | -3.50197 | 4.62E-04 |
| 552 | RHOF | 0.865595 | -0.14434 | -0.22515 | -0.06352 | -3.50058 | 4.64E-04 |
| 553 | CR2 | 0.919609 | -0.08381 | -0.13076 | -0.03686 | -3.49855 | 4.68E-04 |
| 554 | MFNG | 0.832322 | -0.18354 | -0.28643 | -0.08064 | -3.496 | 4.72E-04 |
| 555 | SPNS3 | 0.850684 | -0.16171 | -0.25241 | -0.07102 | -3.49481 | 4.74E-04 |
| 556 | FCHO1 | 0.864974 | -0.14506 | -0.22663 | -0.06348 | -3.4852 | 4.92E-04 |
| 557 | CCDC69 | 0.857063 | -0.15424 | -0.24108 | -0.06741 | -3.48146 | 4.99E-04 |
| 558 | PTPRO | 0.860762 | -0.14994 | -0.23446 | -0.06542 | -3.47701 | 5.07E-04 |
| 559 | SLC2A5 | 0.874858 | -0.13369 | -0.20912 | -0.05827 | -3.47419 | 5.12E-04 |
| 560 | GAPT | 0.870401 | -0.1388 | -0.21712 | -0.06048 | -3.47335 | 5.14E-04 |
| 561 | TNFRSF13B | 0.883175 | -0.12423 | -0.19453 | -0.05393 | -3.46347 | 5.33E-04 |
| 562 | P2RX1 | 0.873796 | -0.13491 | -0.21133 | -0.05848 | -3.45979 | 5.41E-04 |
| 563 | TNFSF8 | 0.861191 | -0.14944 | -0.23416 | -0.06472 | -3.45733 | 5.46E-04 |
| 564 | HCK | 0.872841 | -0.136 | -0.21315 | -0.05885 | -3.45502 | 5.50E-04 |
| 565 | VMO1 | 0.849984 | -0.16254 | -0.25481 | -0.07027 | -3.45259 | 5.55E-04 |
| 566 | FAM49A | 0.867053 | -0.14265 | -0.22364 | -0.06167 | -3.45251 | 5.55E-04 |
| 567 | PACSIN1 | 0.863507 | -0.14675 | -0.23014 | -0.06336 | -3.44921 | 5.62E-04 |
| 568 | C1orf54 | 0.793912 | -0.23078 | -0.36197 | -0.09959 | -3.44786 | 5.65E-04 |
| 569 | FGD3 | 0.854527 | -0.15721 | -0.24658 | -0.06783 | -3.44753 | 5.66E-04 |
| 570 | NCR1 | 0.823283 | -0.19446 | -0.30561 | -0.0833 | -3.42867 | 6.07E-04 |
| 571 | SIGLEC9 | 0.856925 | -0.15441 | -0.24277 | -0.06604 | -3.4247 | 6.15E-04 |
| 572 | TMEM155 | 0.858554 | -0.15251 | -0.23982 | -0.06519 | -3.42328 | 6.19E-04 |
| 573 | ITM2A | 0.87518 | -0.13333 | -0.20983 | -0.05682 | -3.41549 | 6.37E-04 |
| 574 | GPR77 | 0.806324 | -0.21527 | -0.33897 | -0.09157 | -3.41092 | 6.47E-04 |
| 575 | KIAA1324 | 0.857653 | -0.15356 | -0.24185 | -0.06526 | -3.40876 | 6.53E-04 |
| 576 | ARHGAP27 | 0.811443 | -0.20894 | -0.32939 | -0.08849 | -3.39995 | 6.74E-04 |
| 577 | LAIR2 | 0.836992 | -0.17794 | -0.28054 | -0.07534 | -3.39918 | 6.76E-04 |
| 578 | KYNU | 0.887593 | -0.11924 | -0.18802 | -0.05046 | -3.39788 | 6.79E-04 |
| 579 | TAS1R3 | 0.842163 | -0.17178 | -0.27101 | -0.07255 | -3.39295 | 6.91E-04 |
| 580 | ITGAM | 0.856458 | -0.15495 | -0.24447 | -0.06544 | -3.39269 | 6.92E-04 |
| 581 | TYROBP | 0.862746 | -0.14764 | -0.23296 | -0.06231 | -3.39121 | 6.96E-04 |
| 582 | SIGLEC7 | 0.86537 | -0.1446 | -0.22818 | -0.06102 | -3.39085 | 6.97E-04 |
| 583 | LOC100130872 | 0.859174 | -0.15178 | -0.23953 | -0.06403 | -3.39016 | 6.99E-04 |
| 584 | SLC9A9 | 0.842511 | -0.17137 | -0.27059 | -0.07215 | -3.38526 | 7.11E-04 |
| 585 | DUSP26 | 0.82827 | -0.18842 | -0.29751 | -0.07932 | -3.38499 | 7.12E-04 |
| 586 | INSL3 | 0.815046 | -0.20451 | -0.32294 | -0.08608 | -3.38452 | 7.13E-04 |
| 587 | CAMK4 | 0.885846 | -0.12121 | -0.19146 | -0.05097 | -3.38206 | 7.19E-04 |
| 588 | CCDC152 | 0.872071 | -0.13688 | -0.21624 | -0.05753 | -3.38092 | 7.22E-04 |
| 589 | MACC1 | 0.859549 | -0.15135 | -0.23946 | -0.06323 | -3.36643 | 7.61E-04 |
| 590 | RARRES1 | 0.87539 | -0.13309 | -0.21062 | -0.05555 | -3.36427 | 7.67E-04 |
| 591 | FBP1 | 0.875998 | -0.13239 | -0.20958 | -0.0552 | -3.36167 | 7.75E-04 |
| 592 | BCL11B | 0.895382 | -0.1105 | -0.17501 | -0.046 | -3.35754 | 7.86E-04 |
| 593 | SLC40A1 | 0.875662 | -0.13278 | -0.21051 | -0.05504 | -3.34777 | 8.15E-04 |
| 594 | COL4A4 | 0.872325 | -0.13659 | -0.21662 | -0.05656 | -3.34521 | 8.22E-04 |
| 595 | MLXIPL | 0.88021 | -0.12759 | -0.20246 | -0.05273 | -3.34027 | 8.37E-04 |
| 596 | DAPK2 | 0.836805 | -0.17816 | -0.28273 | -0.0736 | -3.33957 | 8.39E-04 |
| 597 | RAC2 | 0.88601 | -0.12103 | -0.19207 | -0.04999 | -3.33902 | 8.41E-04 |
| 598 | CD180 | 0.874126 | -0.13453 | -0.2135 | -0.05556 | -3.33883 | 8.41E-04 |
| 599 | LILRA5 | 0.866529 | -0.14326 | -0.22738 | -0.05914 | -3.33789 | 8.44E-04 |
| 600 | TCL1A | 0.909472 | -0.09489 | -0.15061 | -0.03917 | -3.33781 | 8.44E-04 |
| 601 | GPA33 | 0.812455 | -0.20769 | -0.32981 | -0.08558 | -3.33348 | 8.58E-04 |
| 602 | ASGR2 | 0.84565 | -0.16765 | -0.26634 | -0.06896 | -3.32935 | 8.70E-04 |
| 603 | CD52 | 0.891456 | -0.1149 | -0.18268 | -0.04712 | -3.32261 | 8.92E-04 |
| 604 | NAPSB | 0.905921 | -0.0988 | -0.15728 | -0.04032 | -3.31142 | 9.28E-04 |
| 605 | CD163 | 0.885283 | -0.12185 | -0.19418 | -0.04952 | -3.30184 | 9.61E-04 |
| 606 | BEND4 | 0.860717 | -0.14999 | -0.2391 | -0.06088 | -3.29899 | 9.70E-04 |
| 607 | CXCR5 | 0.905484 | -0.09929 | -0.1583 | -0.04027 | -3.29735 | 9.76E-04 |
| 608 | FOXP3 | 0.866803 | -0.14294 | -0.22804 | -0.05785 | -3.29238 | 9.93E-04 |
| 609 | SLAMF7 | 0.910455 | -0.09381 | -0.14967 | -0.03795 | -3.29172 | 9.96E-04 |
| 610 | BTK | 0.867816 | -0.14178 | -0.22624 | -0.05731 | -3.28981 | 0.001003 |
| 611 | BLK | 0.901668 | -0.10351 | -0.16519 | -0.04183 | -3.28932 | 0.001004 |
| 612 | GTSF1 | 0.915001 | -0.08883 | -0.14183 | -0.03583 | -3.28479 | 0.001021 |
| 613 | HAMP | 0.895904 | -0.10992 | -0.17556 | -0.04428 | -3.28217 | 0.00103 |
| 614 | STX11 | 0.85442 | -0.15733 | -0.25146 | -0.06321 | -3.27609 | 0.001053 |
| 615 | CFP | 0.868303 | -0.14121 | -0.22594 | -0.05649 | -3.26685 | 0.001088 |
| 616 | FGR | 0.865012 | -0.14501 | -0.23204 | -0.05798 | -3.2657 | 0.001092 |
| 617 | JAKMIP2 | 0.893395 | -0.11273 | -0.18052 | -0.04494 | -3.25918 | 0.001117 |
| 618 | PIK3R5 | 0.862131 | -0.14835 | -0.2376 | -0.05909 | -3.25754 | 0.001124 |
| 619 | RHOU | 0.844907 | -0.16853 | -0.27019 | -0.06686 | -3.24902 | 0.001158 |
| 620 | PARP8 | 0.878732 | -0.12928 | -0.20766 | -0.05089 | -3.2323 | 0.001228 |
| 621 | SKAP1 | 0.907116 | -0.09748 | -0.15668 | -0.03829 | -3.2275 | 0.001249 |
| 622 | IL18R1 | 0.870637 | -0.13853 | -0.2227 | -0.05437 | -3.22598 | 0.001255 |
| 623 | SERPINA1 | 0.899838 | -0.10554 | -0.16968 | -0.0414 | -3.22509 | 0.001259 |
| 624 | RBM47 | 0.863567 | -0.14668 | -0.23594 | -0.05742 | -3.22091 | 0.001278 |
| 625 | LYN | 0.837792 | -0.17699 | -0.28485 | -0.06912 | -3.21597 | 0.0013 |
| 626 | C19orf35 | 0.832831 | -0.18292 | -0.29458 | -0.07127 | -3.21097 | 0.001323 |
| 627 | VNN1 | 0.865345 | -0.14463 | -0.23306 | -0.05619 | -3.20531 | 0.001349 |
| 628 | SIGLEC8 | 0.904203 | -0.1007 | -0.16262 | -0.03878 | -3.18739 | 0.001436 |
| 629 | CSF2RA | 0.891902 | -0.1144 | -0.1851 | -0.04369 | -3.17116 | 0.001518 |
| 630 | CD40LG | 0.882326 | -0.12519 | -0.20261 | -0.04778 | -3.16948 | 0.001527 |
| 631 | TMC8 | 0.875341 | -0.13314 | -0.21563 | -0.05065 | -3.16331 | 0.00156 |
| 632 | TMEM176A | 0.862943 | -0.14741 | -0.2388 | -0.05601 | -3.16121 | 0.001571 |
| 633 | KCNMB1 | 0.844426 | -0.1691 | -0.27395 | -0.06425 | -3.16096 | 0.001573 |
| 634 | CCRL2 | 0.852014 | -0.16015 | -0.2595 | -0.06081 | -3.1596 | 0.00158 |
| 635 | GPR120 | 0.810855 | -0.20967 | -0.33998 | -0.07935 | -3.15342 | 0.001614 |
| 636 | CCR7 | 0.907861 | -0.09666 | -0.15675 | -0.03658 | -3.15327 | 0.001615 |
| 637 | ABCG1 | 0.857997 | -0.15315 | -0.24873 | -0.05757 | -3.14058 | 0.001686 |
| 638 | DGKA | 0.834509 | -0.18091 | -0.29391 | -0.06792 | -3.13805 | 0.001701 |
| 639 | AXL | 0.874638 | -0.13394 | -0.21776 | -0.05013 | -3.1322 | 0.001735 |
| 640 | CXCR4 | 0.857929 | -0.15323 | -0.24918 | -0.05729 | -3.13027 | 0.001746 |
| 641 | BTG2 | 0.823967 | -0.19362 | -0.31489 | -0.07236 | -3.12959 | 0.00175 |
| 642 | CD244 | 0.883554 | -0.1238 | -0.20139 | -0.04621 | -3.12738 | 0.001764 |
| 643 | LILRB3 | 0.864625 | -0.14546 | -0.23668 | -0.05424 | -3.12546 | 0.001775 |
| 644 | FAM107B | 0.855006 | -0.15665 | -0.25495 | -0.05835 | -3.12327 | 0.001789 |
| 645 | SAMD12 | 0.881981 | -0.12559 | -0.20448 | -0.04669 | -3.11987 | 0.001809 |
| 646 | CXCL16 | 0.836495 | -0.17853 | -0.29071 | -0.06636 | -3.11942 | 0.001812 |
| 647 | PRR5L | 0.815386 | -0.20409 | -0.33258 | -0.0756 | -3.11322 | 0.001851 |
| 648 | ATP2A3 | 0.878341 | -0.12972 | -0.21142 | -0.04802 | -3.11199 | 0.001858 |
| 649 | FAM20A | 0.876777 | -0.1315 | -0.21448 | -0.04852 | -3.10607 | 0.001896 |
| 650 | LGALS9C | 0.847523 | -0.16544 | -0.26993 | -0.06095 | -3.10316 | 0.001915 |
| 651 | NLRP3 | 0.84685 | -0.16623 | -0.27138 | -0.06109 | -3.09868 | 0.001944 |
| 652 | SLCO2B1 | 0.885022 | -0.12214 | -0.19946 | -0.04483 | -3.09635 | 0.001959 |
| 653 | CACNA1I | 0.874766 | -0.1338 | -0.21849 | -0.0491 | -3.0963 | 0.00196 |
| 654 | FPR1 | 0.88141 | -0.12623 | -0.20624 | -0.04623 | -3.09242 | 0.001985 |
| 655 | SPIB | 0.905981 | -0.09874 | -0.16151 | -0.03597 | -3.08311 | 0.002048 |
| 656 | GATA3 | 0.894571 | -0.11141 | -0.18224 | -0.04058 | -3.08307 | 0.002049 |
| 657 | NLRP7 | 0.859678 | -0.1512 | -0.2475 | -0.05489 | -3.07711 | 0.00209 |
| 658 | NCF2 | 0.874959 | -0.13358 | -0.21866 | -0.04849 | -3.07698 | 0.002091 |
| 659 | RASGRP2 | 0.888615 | -0.11809 | -0.19339 | -0.04279 | -3.07369 | 0.002114 |
| 660 | ELMO1 | 0.865509 | -0.14444 | -0.23665 | -0.05223 | -3.07005 | 0.00214 |
| 661 | VSIG4 | 0.883699 | -0.12364 | -0.20282 | -0.04445 | -3.06024 | 0.002212 |
| 662 | CARD9 | 0.867871 | -0.14171 | -0.23252 | -0.0509 | -3.05858 | 0.002224 |
| 663 | CLDN11 | 0.917708 | -0.08588 | -0.14092 | -0.03083 | -3.05771 | 0.00223 |
| 664 | PTAFR | 0.884946 | -0.12223 | -0.20081 | -0.04364 | -3.04847 | 0.0023 |
| 665 | HVCN1 | 0.865175 | -0.14482 | -0.23798 | -0.05166 | -3.04688 | 0.002312 |
| 666 | C17orf28 | 0.866793 | -0.14296 | -0.23496 | -0.05095 | -3.04529 | 0.002325 |
| 667 | TC2N | 0.904038 | -0.10088 | -0.16584 | -0.03592 | -3.04385 | 0.002336 |
| 668 | CCR4 | 0.88472 | -0.12248 | -0.20153 | -0.04344 | -3.03696 | 0.00239 |
| 669 | KLHL6 | 0.881263 | -0.1264 | -0.20799 | -0.04481 | -3.03636 | 0.002394 |
| 670 | RASGEF1B | 0.85996 | -0.15087 | -0.24831 | -0.05343 | -3.03474 | 0.002407 |
| 671 | CD300A | 0.869563 | -0.13976 | -0.23004 | -0.04949 | -3.03454 | 0.002409 |
| 672 | NTNG2 | 0.869738 | -0.13956 | -0.2298 | -0.04932 | -3.03124 | 0.002436 |
| 673 | PKHD1L1 | 0.861889 | -0.14863 | -0.24477 | -0.05249 | -3.02991 | 0.002446 |
| 674 | IL3RA | 0.831298 | -0.18477 | -0.30431 | -0.06522 | -3.02922 | 0.002452 |
| 675 | XCR1 | 0.852943 | -0.15906 | -0.262 | -0.05612 | -3.02852 | 0.002458 |
| 676 | CD1B | 0.858641 | -0.1524 | -0.25123 | -0.05358 | -3.02251 | 0.002507 |
| 677 | FCER2 | 0.914477 | -0.0894 | -0.14763 | -0.03117 | -3.00912 | 0.00262 |
| 678 | SEPP1 | 0.88693 | -0.11999 | -0.19829 | -0.04169 | -3.00336 | 0.00267 |
| 679 | TNFRSF8 | 0.877011 | -0.13124 | -0.21728 | -0.04519 | -2.98936 | 0.002796 |
| 680 | CSF3R | 0.880026 | -0.1278 | -0.21173 | -0.04388 | -2.9846 | 0.00284 |
| 681 | CD1C | 0.896202 | -0.10959 | -0.1816 | -0.03758 | -2.98269 | 0.002857 |
| 682 | TOX2 | 0.889051 | -0.1176 | -0.19492 | -0.04028 | -2.98107 | 0.002872 |
| 683 | C11orf75 | 0.8373 | -0.17757 | -0.29441 | -0.06074 | -2.97888 | 0.002893 |
| 684 | CLIC5 | 0.856603 | -0.15478 | -0.2567 | -0.05286 | -2.97639 | 0.002917 |
| 685 | C14orf64 | 0.871418 | -0.13763 | -0.22834 | -0.04693 | -2.97391 | 0.00294 |
| 686 | JAK3 | 0.880371 | -0.12741 | -0.2118 | -0.04303 | -2.95929 | 0.003084 |
| 687 | CNR2 | 0.879657 | -0.12822 | -0.21318 | -0.04326 | -2.95801 | 0.003096 |
| 688 | FAM129C | 0.8907 | -0.11575 | -0.19255 | -0.03895 | -2.95384 | 0.003138 |
| 689 | CCL3 | 0.87595 | -0.13245 | -0.22039 | -0.0445 | -2.95171 | 0.00316 |
| 690 | AKNA | 0.84032 | -0.17397 | -0.28956 | -0.05839 | -2.95 | 0.003178 |
| 691 | FMNL1 | 0.880272 | -0.12752 | -0.21226 | -0.04279 | -2.94965 | 0.003181 |
| 692 | TNFAIP2 | 0.860184 | -0.15061 | -0.25078 | -0.05044 | -2.94686 | 0.00321 |
| 693 | SLC15A3 | 0.875598 | -0.13285 | -0.2215 | -0.0442 | -2.93711 | 0.003313 |
| 694 | PDE6B | 0.877339 | -0.13086 | -0.21855 | -0.04317 | -2.92488 | 0.003446 |
| 695 | ITGA4 | 0.905227 | -0.09957 | -0.16629 | -0.03284 | -2.92471 | 0.003448 |
| 696 | SNAI3 | 0.845559 | -0.16776 | -0.2808 | -0.05471 | -2.90863 | 0.00363 |
| 697 | TNFRSF11B | 0.903848 | -0.10109 | -0.16969 | -0.0325 | -2.88868 | 0.003869 |
| 698 | CCL19 | 0.938242 | -0.06375 | -0.10704 | -0.02045 | -2.88598 | 0.003902 |
| 699 | CCL23 | 0.854645 | -0.15707 | -0.26393 | -0.05021 | -2.88078 | 0.003967 |
| 700 | CYFIP2 | 0.880598 | -0.12715 | -0.21373 | -0.04058 | -2.87878 | 0.003992 |
| 701 | C10orf105 | 0.85436 | -0.1574 | -0.265 | -0.04981 | -2.86721 | 0.004141 |
| 702 | ITGAD | 0.895714 | -0.11013 | -0.18546 | -0.0348 | -2.86546 | 0.004164 |
| 703 | MAP4K1 | 0.889044 | -0.11761 | -0.19816 | -0.03706 | -2.86157 | 0.004215 |
| 704 | ANO9 | 0.91017 | -0.09412 | -0.15878 | -0.02946 | -2.8531 | 0.004329 |
| 705 | MMP25 | 0.879982 | -0.12785 | -0.21624 | -0.03947 | -2.83518 | 0.00458 |
| 706 | AMPD3 | 0.868996 | -0.14042 | -0.23749 | -0.04334 | -2.83509 | 0.004581 |
| 707 | RSPO3 | 0.893738 | -0.11234 | -0.19009 | -0.0346 | -2.83219 | 0.004623 |
| 708 | C12orf59 | 0.871666 | -0.13735 | -0.23258 | -0.04212 | -2.82688 | 0.0047 |
| 709 | RNASE2 | 0.876502 | -0.13182 | -0.22345 | -0.04018 | -2.81938 | 0.004812 |
| 710 | FPR3 | 0.896452 | -0.10931 | -0.18552 | -0.0331 | -2.81139 | 0.004933 |
| 711 | OSCAR | 0.881023 | -0.12667 | -0.21509 | -0.03825 | -2.8079 | 0.004987 |
| 712 | DERL3 | 0.898825 | -0.10667 | -0.18118 | -0.03216 | -2.8058 | 0.005019 |
| 713 | RGS9 | 0.883679 | -0.12366 | -0.21013 | -0.0372 | -2.80317 | 0.00506 |
| 714 | CD209 | 0.889543 | -0.11705 | -0.199 | -0.0351 | -2.79938 | 0.00512 |
| 715 | LPAR5 | 0.871255 | -0.13782 | -0.23461 | -0.04103 | -2.79086 | 0.005257 |
| 716 | CHI3L2 | 0.917047 | -0.0866 | -0.14745 | -0.02575 | -2.7893 | 0.005282 |
| 717 | PSTPIP2 | 0.882935 | -0.1245 | -0.21225 | -0.03676 | -2.78096 | 0.00542 |
| 718 | CCDC141 | 0.896759 | -0.10897 | -0.18584 | -0.03209 | -2.77821 | 0.005466 |
| 719 | CHI3L1 | 0.926725 | -0.0761 | -0.12989 | -0.02231 | -2.77269 | 0.00556 |
| 720 | GPR55 | 0.891235 | -0.11515 | -0.19679 | -0.03351 | -2.76436 | 0.005703 |
| 721 | C1R | 0.882735 | -0.12473 | -0.21321 | -0.03625 | -2.76295 | 0.005728 |
| 722 | KCNK13 | 0.874167 | -0.13448 | -0.22996 | -0.039 | -2.76057 | 0.00577 |
| 723 | CLEC17A | 0.891764 | -0.11455 | -0.19638 | -0.03273 | -2.74381 | 0.006073 |
| 724 | PPP1R16B | 0.888119 | -0.11865 | -0.20344 | -0.03386 | -2.74259 | 0.006096 |
| 725 | UCP2 | 0.869932 | -0.13934 | -0.23917 | -0.03951 | -2.73569 | 0.006225 |
| 726 | LSP1 | 0.889996 | -0.11654 | -0.20011 | -0.03296 | -2.73304 | 0.006275 |
| 727 | TMEM130 | 0.877947 | -0.13017 | -0.22371 | -0.03663 | -2.72755 | 0.006381 |
| 728 | SIDT1 | 0.888114 | -0.11865 | -0.20393 | -0.03338 | -2.7273 | 0.006386 |
| 729 | IFI30 | 0.884906 | -0.12227 | -0.21032 | -0.03423 | -2.72182 | 0.006492 |
| 730 | CFH | 0.881298 | -0.12636 | -0.21736 | -0.03536 | -2.72152 | 0.006498 |
| 731 | LY86 | 0.888436 | -0.11829 | -0.20357 | -0.03301 | -2.71867 | 0.006555 |
| 732 | ICAM2 | 0.856513 | -0.15489 | -0.26675 | -0.04302 | -2.71377 | 0.006652 |
| 733 | CMKLR1 | 0.889404 | -0.1172 | -0.20198 | -0.03242 | -2.70957 | 0.006737 |
| 734 | ADAM8 | 0.890006 | -0.11653 | -0.20086 | -0.0322 | -2.7083 | 0.006763 |
| 735 | CD70 | 0.904446 | -0.10043 | -0.17314 | -0.02773 | -2.70752 | 0.006779 |
| 736 | TNFSF12-TNFSF13 | 0.917778 | -0.0858 | -0.14803 | -0.02357 | -2.70211 | 0.00689 |
| 737 | APOB48R | 0.872605 | -0.13627 | -0.23514 | -0.0374 | -2.70138 | 0.006905 |
| 738 | VPREB3 | 0.905217 | -0.09958 | -0.17196 | -0.0272 | -2.69649 | 0.007007 |
| 739 | RBP5 | 0.910697 | -0.09355 | -0.16177 | -0.02532 | -2.68738 | 0.007201 |
| 740 | OTOF | 0.874893 | -0.13365 | -0.23154 | -0.03577 | -2.67615 | 0.007447 |
| 741 | PLA2G7 | 0.90071 | -0.10457 | -0.18148 | -0.02766 | -2.66493 | 0.0077 |
| 742 | C1S | 0.897139 | -0.10854 | -0.18851 | -0.02858 | -2.66048 | 0.007803 |
| 743 | C3 | 0.932495 | -0.06989 | -0.1214 | -0.01838 | -2.65935 | 0.007829 |
| 744 | FMO2 | 0.91057 | -0.09368 | -0.16281 | -0.02456 | -2.65635 | 0.007899 |
| 745 | LILRB5 | 0.893092 | -0.11307 | -0.19658 | -0.02955 | -2.65337 | 0.007969 |
| 746 | ABCC3 | 0.904656 | -0.1002 | -0.17426 | -0.02614 | -2.65169 | 0.008009 |
| 747 | NMUR1 | 0.87481 | -0.13375 | -0.23321 | -0.03429 | -2.63573 | 0.008396 |
| 748 | LILRA4 | 0.91133 | -0.09285 | -0.1625 | -0.0232 | -2.61273 | 0.008982 |
| 749 | CCDC88C | 0.887949 | -0.11884 | -0.20824 | -0.02944 | -2.60549 | 0.009174 |
| 750 | LBH | 0.863812 | -0.1464 | -0.25665 | -0.03615 | -2.60273 | 0.009248 |
| 751 | TGM2 | 0.879636 | -0.12825 | -0.22508 | -0.03141 | -2.59578 | 0.009438 |
| 752 | SIGLEC6 | 0.850133 | -0.16236 | -0.28519 | -0.03953 | -2.59073 | 0.009577 |
| 753 | EPHB2 | 0.890149 | -0.11637 | -0.20448 | -0.02825 | -2.58829 | 0.009645 |
| 754 | SLC38A1 | 0.923756 | -0.07931 | -0.13961 | -0.01901 | -2.57768 | 0.009947 |
| 755 | STK17B | 0.881418 | -0.12622 | -0.22223 | -0.03021 | -2.57672 | 0.009974 |
|  |  |  |  |  |  |  |  |
