# Supplementary material for: Data-driven analysis to identify prognostic immune-related biomarkers in BRAF mutated cutaneous melanoma microenvironment
Source: Front Genet. 2022 Nov 30;13:1081418. doi: 10.3389/fgene.2022.1081418 (PMC9749953; doi:10.3389/fgene.2022.1081418)
Supplement: Supplementary file 2 [file Table2.DOC]

**Supplementary Table 2. The verified genes associated with overall survival in GEO database (P<0.05)**

|  | Gene ID | HR[exp(coef)] | coef | 95% CI lower | 95% CI upper | Z | P value |
| --- | --- | --- | --- | --- | --- | --- | --- |
| 1 | HLA-DQA1 | 0.716346 | -0.33359 | -0.5444 | -0.12278 | -3.10153 | 0.001925 |
| 2 | HLA-DRA | 0.699425 | -0.3575 | -0.58458 | -0.13041 | -3.08552 | 0.002032 |
| 3 | LAPTM5 | 0.629669 | -0.46256 | -0.76209 | -0.16304 | -3.0268 | 0.002472 |
| 4 | HLA-DPA1 | 0.700474 | -0.356 | -0.5882 | -0.12379 | -3.00487 | 0.002657 |
| 5 | ITM2A | 1.84158 | 0.610624 | 0.211026 | 1.010222 | 2.995014 | 0.002744 |
| 6 | SLCO2B1 | 0.596359 | -0.51691 | -0.85708 | -0.17674 | -2.97829 | 0.002899 |
| 7 | CD74 | 0.713095 | -0.33814 | -0.56584 | -0.11044 | -2.91056 | 0.003608 |
| 8 | HLA-DMA | 0.667197 | -0.40467 | -0.67846 | -0.13088 | -2.89689 | 0.003769 |
| 9 | CD40 | 0.413952 | -0.88201 | -1.48776 | -0.27625 | -2.8538 | 0.00432 |
| 10 | C1QC | 0.703336 | -0.35192 | -0.60407 | -0.09977 | -2.73545 | 0.006229 |
| 11 | IFI30 | 0.654711 | -0.42356 | -0.72899 | -0.11813 | -2.71804 | 0.006567 |
| 12 | CD14 | 0.699981 | -0.3567 | -0.61694 | -0.09646 | -2.68647 | 0.007221 |
| 13 | RASSF4 | 0.522048 | -0.65 | -1.1341 | -0.16589 | -2.63158 | 0.008499 |
| 14 | FGF1 | 0.001297 | -6.64782 | -11.6169 | -1.67872 | -2.6221 | 0.008739 |
| 15 | MS4A7 | 0.504599 | -0.68399 | -1.19604 | -0.17194 | -2.61808 | 0.008843 |
| 16 | MS4A6A | 0.660183 | -0.41524 | -0.72681 | -0.10366 | -2.61204 | 0.009 |
| 17 | C1QA | 0.678393 | -0.38803 | -0.67943 | -0.09663 | -2.60989 | 0.009057 |
| 18 | HSD11B1 | 0.424965 | -0.85575 | -1.50591 | -0.20559 | -2.57975 | 0.009887 |
| 19 | HLA-DMB | 0.720614 | -0.32765 | -0.57709 | -0.07821 | -2.57449 | 0.010039 |
| 20 | CD163 | 0.687468 | -0.37474 | -0.66566 | -0.08382 | -2.52464 | 0.011582 |
| 21 | RARRES1 | 0.561474 | -0.57719 | -1.02782 | -0.12656 | -2.51043 | 0.012058 |
| 22 | CR1 | 0.028246 | -3.5668 | -6.37082 | -0.76277 | -2.49313 | 0.012662 |
| 23 | LYN | 0.682342 | -0.38222 | -0.6847 | -0.07975 | -2.47674 | 0.013259 |
| 24 | KLRC1 | 0.027556 | -3.59152 | -6.44177 | -0.74127 | -2.46969 | 0.013523 |
| 25 | KIR3DL2 | 0.022797 | -3.78113 | -6.8022 | -0.76007 | -2.45307 | 0.014164 |
| 26 | APOL1 | 0.111757 | -2.19143 | -3.94334 | -0.43952 | -2.45168 | 0.014219 |
| 27 | C1QB | 0.750724 | -0.28672 | -0.51609 | -0.05734 | -2.44995 | 0.014287 |
| 28 | CCR1 | 0.400851 | -0.91417 | -1.64934 | -0.17899 | -2.43715 | 0.014804 |
| 29 | AIF1 | 0.680241 | -0.38531 | -0.69689 | -0.07372 | -2.42371 | 0.015363 |
| 30 | PLEK | 0.718031 | -0.33124 | -0.59949 | -0.06299 | -2.42024 | 0.01551 |
| 31 | KIR2DL4 | 0.4202 | -0.86703 | -1.56918 | -0.16487 | -2.42017 | 0.015513 |
| 32 | IGSF6 | 0.543036 | -0.61058 | -1.10886 | -0.1123 | -2.40169 | 0.016319 |
| 33 | HLA-DRB6 | 0.689929 | -0.37117 | -0.67594 | -0.06639 | -2.38693 | 0.01699 |
| 34 | FCGR1A | 0.498887 | -0.69537 | -1.2717 | -0.11905 | -2.36482 | 0.018039 |
| 35 | FAM49A | 0.10421 | -2.26135 | -4.13778 | -0.38492 | -2.36202 | 0.018176 |
| 36 | VNN1 | 0.035723 | -3.33197 | -6.10639 | -0.55755 | -2.35384 | 0.018581 |
| 37 | TGM2 | 0.688887 | -0.37268 | -0.68327 | -0.06208 | -2.35172 | 0.018687 |
| 38 | ABCG1 | 4.284669 | 1.455043 | 0.238392 | 2.671694 | 2.344002 | 0.019078 |
| 39 | ITGB2 | 0.721773 | -0.32604 | -0.59917 | -0.05292 | -2.33969 | 0.0193 |
| 40 | IL33 | 0.58356 | -0.53861 | -0.99091 | -0.0863 | -2.33394 | 0.019599 |
| 41 | IL10 | 0.002995 | -5.81093 | -10.696 | -0.92582 | -2.33141 | 0.019732 |
| 42 | PTAFR | 0.323407 | -1.12884 | -2.07897 | -0.17872 | -2.32863 | 0.019879 |
| 43 | CD80 | 0.165495 | -1.79882 | -3.31345 | -0.28418 | -2.3277 | 0.019928 |
| 44 | LYZ | 0.790878 | -0.23461 | -0.43283 | -0.03639 | -2.31976 | 0.020354 |
| 45 | TLR8 | 0.551904 | -0.59438 | -1.09885 | -0.08991 | -2.30927 | 0.020928 |
| 46 | CCL8 | 0.755083 | -0.28093 | -0.52006 | -0.04179 | -2.30249 | 0.021308 |
| 47 | CYBB | 0.657028 | -0.42003 | -0.77896 | -0.0611 | -2.2936 | 0.021814 |
| 48 | CD72 | 0.531459 | -0.63213 | -1.17262 | -0.09164 | -2.29227 | 0.02189 |
| 49 | PIM2 | 0.728237 | -0.31713 | -0.58885 | -0.0454 | -2.28745 | 0.02217 |
| 50 | HLA-DOA | 0.71068 | -0.34153 | -0.63426 | -0.04881 | -2.28678 | 0.022209 |
| 51 | CD84 | 0.501904 | -0.68935 | -1.28231 | -0.09638 | -2.27853 | 0.022695 |
| 52 | LCP1 | 0.785662 | -0.24123 | -0.44881 | -0.03365 | -2.27771 | 0.022744 |
| 53 | COL4A4 | 0.0897 | -2.41129 | -4.4866 | -0.33598 | -2.27727 | 0.02277 |
| 54 | NCF4 | 0.574585 | -0.55411 | -1.03573 | -0.07249 | -2.25496 | 0.024136 |
| 55 | SLC15A3 | 0.724332 | -0.3225 | -0.60286 | -0.04215 | -2.25465 | 0.024156 |
| 56 | C1orf162 | 0.709715 | -0.34289 | -0.64193 | -0.04385 | -2.24736 | 0.024617 |
| 57 | APOL3 | 0.695865 | -0.3626 | -0.67956 | -0.04564 | -2.2422 | 0.024949 |
| 58 | TYROBP | 0.755377 | -0.28054 | -0.52609 | -0.03498 | -2.23919 | 0.025143 |
| 59 | SIGLEC10 | 0.666938 | -0.40506 | -0.76022 | -0.0499 | -2.23534 | 0.025395 |
| 60 | TNFSF13B | 0.712905 | -0.33841 | -0.63605 | -0.04077 | -2.22841 | 0.025853 |
| 61 | MYO7A | 0.360732 | -1.01962 | -1.91757 | -0.12167 | -2.22553 | 0.026046 |
| 62 | IL18BP | 0.742602 | -0.29759 | -0.55982 | -0.03537 | -2.22431 | 0.026127 |
| 63 | IL2RA | 0.384516 | -0.95577 | -1.80266 | -0.10888 | -2.21194 | 0.026971 |
| 64 | LILRB3 | 0.694659 | -0.36433 | -0.68872 | -0.03995 | -2.20132 | 0.027713 |
| 65 | CFB | 0.648643 | -0.43287 | -0.81926 | -0.04648 | -2.19575 | 0.02811 |
| 66 | ANKRD22 | 0.525087 | -0.64419 | -1.22008 | -0.0683 | -2.19242 | 0.028349 |
| 67 | GPR84 | 0.375027 | -0.98076 | -1.8614 | -0.10012 | -2.18279 | 0.029051 |
| 68 | ARHGAP25 | 0.608767 | -0.49632 | -0.94288 | -0.04977 | -2.17839 | 0.029377 |
| 69 | IL15RA | 0.186453 | -1.67958 | -3.19402 | -0.16513 | -2.17367 | 0.02973 |
| 70 | CECR1 | 0.76475 | -0.26821 | -0.51184 | -0.02457 | -2.15764 | 0.030956 |
| 71 | CSF1R | 0.719975 | -0.32854 | -0.62739 | -0.02968 | -2.15464 | 0.03119 |
| 72 | RASSF5 | 0.675663 | -0.39206 | -0.74884 | -0.03528 | -2.15376 | 0.031259 |
| 73 | CD86 | 0.657358 | -0.41953 | -0.80327 | -0.03578 | -2.14271 | 0.032136 |
| 74 | SLC7A7 | 0.711412 | -0.3405 | -0.65259 | -0.02842 | -2.13842 | 0.032483 |
| 75 | HLA-DPB1 | 0.636422 | -0.45189 | -0.86945 | -0.03433 | -2.12112 | 0.033912 |
| 76 | CD38 | 0.676504 | -0.39082 | -0.75237 | -0.02926 | -2.11859 | 0.034125 |
| 77 | CCL5 | 0.826495 | -0.19056 | -0.36718 | -0.01394 | -2.11465 | 0.03446 |
| 78 | VSIG4 | 0.678473 | -0.38791 | -0.74752 | -0.02831 | -2.11424 | 0.034495 |
| 79 | MGC29506 | 0.780865 | -0.24735 | -0.47687 | -0.01784 | -2.11227 | 0.034663 |
| 80 | HLA-B | 0.774473 | -0.25557 | -0.49368 | -0.01746 | -2.10373 | 0.035402 |
| 81 | IRF8 | 0.761053 | -0.27305 | -0.52791 | -0.0182 | -2.09991 | 0.035737 |
| 82 | CASP5 | 0.062977 | -2.76499 | -5.34607 | -0.18391 | -2.09962 | 0.035763 |
| 83 | GNLY | 0.654888 | -0.42329 | -0.81875 | -0.02784 | -2.09794 | 0.035911 |
| 84 | PILRA | 0.450418 | -0.79758 | -1.54784 | -0.04732 | -2.08357 | 0.037199 |
| 85 | LILRB4 | 0.662126 | -0.4123 | -0.8005 | -0.02409 | -2.08161 | 0.037378 |
| 86 | STX11 | 0.643386 | -0.44101 | -0.8577 | -0.02432 | -2.07437 | 0.038045 |
| 87 | LILRB2 | 0.634539 | -0.45486 | -0.88479 | -0.02492 | -2.07359 | 0.038118 |
| 88 | C3AR1 | 0.470145 | -0.75471 | -1.46817 | -0.04126 | -2.07331 | 0.038143 |
| 89 | AMICA1 | 0.61556 | -0.48522 | -0.9441 | -0.02634 | -2.07248 | 0.03822 |
| 90 | HCK | 0.638506 | -0.44862 | -0.87548 | -0.02177 | -2.0599 | 0.039408 |
| 91 | RNASE6 | 0.621532 | -0.47557 | -0.93143 | -0.01971 | -2.04469 | 0.040886 |
| 92 | ALOX5 | 0.751047 | -0.28629 | -0.56148 | -0.01109 | -2.03895 | 0.041455 |
| 93 | SRGN | 0.769233 | -0.26236 | -0.51594 | -0.00878 | -2.02782 | 0.042579 |
| 94 | GZMB | 0.773422 | -0.25693 | -0.50527 | -0.00859 | -2.02775 | 0.042586 |
| 95 | DOCK2 | 0.740403 | -0.30056 | -0.59237 | -0.00875 | -2.01873 | 0.043515 |
| 96 | SLAMF8 | 0.660823 | -0.41427 | -0.81755 | -0.01098 | -2.01335 | 0.044078 |
| 97 | CCL19 | 0.833634 | -0.18196 | -0.35918 | -0.00474 | -2.01236 | 0.044182 |
| 98 | BTK | 0.625859 | -0.46863 | -0.92612 | -0.01114 | -2.00767 | 0.044678 |
| 99 | CD300LF | 0.59711 | -0.51565 | -1.01945 | -0.01186 | -2.0061 | 0.044846 |
| 100 | IL18RAP | 0.4166 | -0.87563 | -1.73303 | -0.01823 | -2.00163 | 0.045324 |
| 101 | IRF1 | 0.760195 | -0.27418 | -0.54273 | -0.00563 | -2.00103 | 0.045389 |
| 102 | CCL3 | 0.651754 | -0.42809 | -0.84911 | -0.00707 | -1.99287 | 0.046276 |
| 103 | TNFAIP2 | 0.518966 | -0.65592 | -1.30181 | -0.01003 | -1.99039 | 0.046548 |
| 104 | UCP2 | 0.729558 | -0.31532 | -0.62758 | -0.00305 | -1.97914 | 0.047801 |
| 105 | SLC2A5 | 0.697068 | -0.36087 | -0.72109 | -0.00066 | -1.96354 | 0.049584 |
| 106 | HAVCR2 | 0.736759 | -0.30549 | -0.61058 | -0.00041 | -1.96262 | 0.049691 |
| 107 | CLEC4A | 0.476869 | -0.74051 | -1.48064 | -0.00038 | -1.96098 | 0.049881 |
|  |  |  |  |  |  |  |  |
